# Heterointeraction-Induced Nucleation Promoting Vertical Growth and Suppressing Phase Separation for Efficient Wide-Bandgap Perovskite Solar Cells and Tandem Devices

**DOI:** 10.34133/research.0892

**Published:** 2025-09-22

**Authors:** Changbo Li, Yuyi Wang, Weiyin Gao, Jianxiong Yang, Zelin Wang, Xiaojia Zhao, Xinyuan Liu, Liangxu Wang, Yile Liu, Xiaobo Wang, He Dong, Long Zhou, Weidong Zhu, Chenxin Ran, Wei Huang

**Affiliations:** ^1^ Frontiers Science Center for Flexible Electronics, Xi’an Institute of Flexible Electronics (IFE) and Northwestern Polytechnical University, Xi’an 710072, China.; ^2^ Engineering Research Center of Smart Energy and Carbon Neutral in Oil & Gas Field Universities of Shaanxi Province, College of New Energy, Xi’an Shiyou University, Xi’an 710065, China.; ^3^ State Key Laboratory of Wide-Bandgap Semiconductor Devices and Integrated Technology, Xidian University, Xi’an 710071, China.; ^4^ Research & Development Institute of Northwestern Polytechnical University in Shenzhen, Shenzhen 518063, China.; ^5^ Chongqing Innovation Center, Northwestern Polytechnical University, Chongqing 401135, China.

## Abstract

As a key component of perovskite-based tandem photovoltaic devices, wide-bandgap (WBG) perovskite solar cells (PSCs) have been extensively explored recently. For WBG perovskite with mixed Cs/formamidinium (FA) cations and I/Br anions, it is challenging to control ordered crystal growth due to component complexity, and defect-mediated halogen migration causes severe phase separation, leading to poor film quality and inferior device performance. In this work, CsPb_2_Br_5_ has been developed to serve as a heteronucleation agent to regulate the crystal growth of Cs_0.2_FA_0.8_Pb(I_0.6_Br_0.4_)_3_ perovskite for producing high-quality film. Theoretical and experimental results show that CsPb_2_Br_5_ reduces the energy barrier of nucleation and increases the defect formation energy, which not only promotes the homogeneous nucleation and guides the vertical growth of perovskite, which is beneficial for charge transport, but also reduces the defect density and releases the residual strains that suppress phase separation in the film. Therefore, optimized 1.80-eV PSCs yield a champion power conversion efficiency of 20.14% and a record-high fill factor of 85.39% with enhanced device stability. Notably, the constructed 4-terminal tandem devices yield promising power conversion efficiencies of 31.13% (perovskite/silicon) and 28.39% (all-perovskite). This work adds critical building blocks for efficient and stable WBG PSCs by rational crystallization control.

## Introduction

Organic–inorganic hybrid lead halide perovskites possess outstanding optoelectronic features and industrialization advantages, enabling them to be ideal candidates as the core material in next-generation optoelectronic devices [1]. Among them, perovskite solar cells (PSCs) have made exhilarating progress, and specifically, single-junction PSCs have achieved certified power conversion efficiencies (PCEs) of 27% on small-area devices (0.1 cm^2^) [2] and over 20% on large-area modules (1 to 100 cm^2^) with a robust life-span [3]. Moreover, the solution-processable deposition of perovskite films is shown to be compatible with various existing large-area thin-film fabrication techniques [4–8], which demonstrates their huge promise in large-scale commercialization [9–11].

Nevertheless, due to the Shockley–Queisser (S-Q) PCE limit theory for solar cells [12], the current single-junction PSCs with a typical bandgap of 1.6 eV suffer from the problem of approaching an S-Q limit PCE of ~31%; in other words, further PCE improvement has hit a bottleneck. To solve this problem, constructing multijunction tandem solar cells based on light harvesters with different bandgaps is a feasible way in principle, and the theoretical PCE of 2-junction tandem solar cells can reach over 44% using the combination of a 1.1- to 1.2-eV narrow-bandgap (NBG) subcell and a 1.7- to 1.8-eV wide-bandgap (WBG) subcell [13]. Following this concept, perovskite-based tandem solar cells, including perovskite/perovskite [14,15], perovskite/silicon [16,17], perovskite/copper indium gallium selenide [18], and perovskite/organic [19] tandems, have been extensively developed during the past few years, and it did not take long for perovskite/silicon and perovskite/perovskite tandems to achieve PCEs of over 33% and 30%, respectively [2,20]. Note that all these perovskite-based tandem devices need a WBG subcell with a bandgap ranging from 1.68 to 1.8 eV according to the type of NBG subcell that they will be paired with [21–23], and thus, it is of great significance to develop efficient WBG PSCs to promote the performance of perovskite-based tandem devices [24–27].

Among WBG perovskites, the methylammonium (MA)-free Cs_0.2_FA_0.8_Pb(I_0.6_Br_0.4_)_3_ one with a bandgap of 1.79 eV has been widely studied, as this bandgap is around the ideal value that can be compatible with various NBG subcells to achieve the maximum PCE of 4-terminal (4T) tandem devices [28]. Currently, research mainly focuses on solving the intrinsic problems of the Cs_0.2_FA_0.8_Pb(I_0.6_Br_0.4_)_3_ perovskite that hinder the realization of efficient WBG PSCs, such as uncontrollable crystallization, photoinduced phase separation, and large open-circuit voltage (*V*_OC_) loss [29]. Inspiringly, the *V*_OC_ loss problem has been well resolved by developing a series of self-assembled monolayers exceptional ability and diverse roles of energy-level regulation, interface modification, defect passivation, and charge transportation [30–32]. However, the critical issues of crystallization control and phase separation still face substantial challenges. So far, a few efforts have been made to address the problems of crystallization control and phase separation by developing functional agents to retard crystallization, regulate crystal distortions, and passivate defects for improving the crystallization quality and phase stability of Cs_0.2_FA_0.8_Pb(I_0.6_Br_0.4_)_3_ perovskite film, such as methylammonium chloride [33], 4-fluoro-phenylethylammonium iodide [34], *trans*-ferulic acid [35], redox mediators [36], ionic salts [37], and organic cations [38,39]. Therefore, rational design of additives to synergistically control crystal growth and suppress defect formation is highly expected to further improve the quality of WBG perovskite films and boost the performance of their PSCs.

In this work, an all-inorganic 2-dimensional CsPb_2_Br_5_ flake was developed to serve as a crystallization regulator for Cs_0.2_FA_0.8_Pb(I_0.6_Br_0.4_)_3_ perovskite. CsPb_2_Br_5_ was found to not only facilitate the heteronucleation of the perovskite on it due to the reduced formation energy of the perovskite structure but also reduce the defect trap-state density of the perovskite film due to the increased defect formation energy. Importantly, CsPb_2_Br_5_ could guide top-down growth to produce perovskite films composed of vertically thorough and laterally tightly arranged crystals with enhanced orientation and released strains, leading to the efficient charge transportation and suppressed phase separation of the film. PSCs based on the target WBG film produced champion PCE of 20.14% and fill factor (FF) of 85.39%, which is the highest value in 1.80-eV PSCs. In addition, the unencapsulated device showed robust illumination and storage stability. By combining a semitransparent (S-T) 1.80-eV WBG device with a heterojunction back contact (HBC) silicon solar cell, the 4T tandem device achieved promising PCEs of 31.13% (perovskite/silicon) and 28.39% (all-perovskite).

## Results and Discussion

**Fig. 1.**
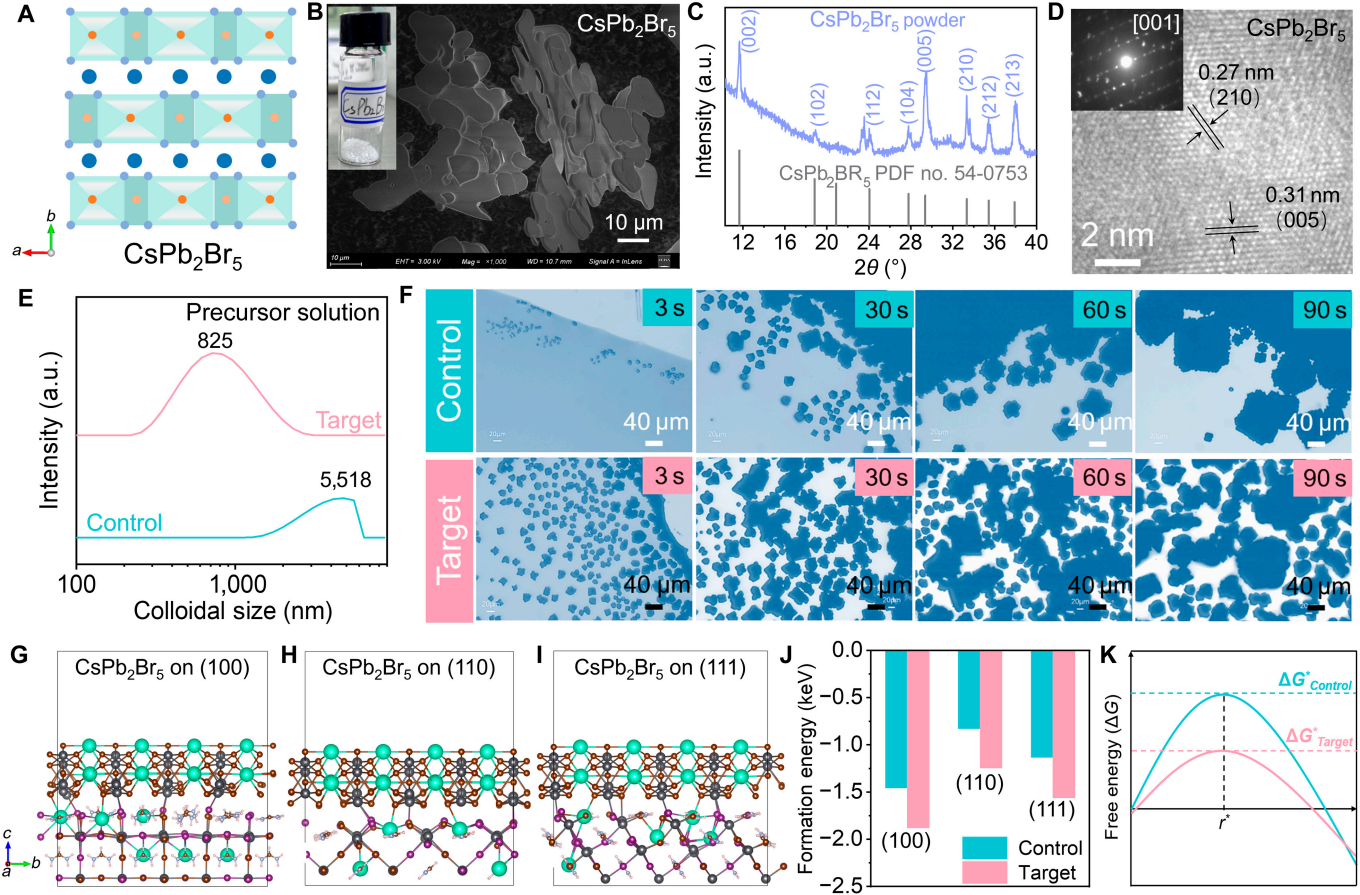
(A) Schematic of the crystal structure of CsPb_2_Br_5_. (B) Scanning electron microscopy (SEM) image and photograph (inset) of CsPb_2_Br_5_ flakes. (C) X-ray diffraction (XRD) pattern of CsPb_2_Br_5_. (D) High-resolution transmission electron microscopy (HRTEM) and selected area electron diffraction (SAED) (inset) of CsPb_2_Br_5_. (E) Dynamic light scattering (DLS) of Cs_0.2_FA_0.8_Pb(I_0.6_Br_0.4_)_3_ precursor solutions without (control) and with (target) CsPb_2_Br_5_. (F) Optical microscopic images of control and target perovskite precursor solution heating for different times. Optimized crystal structures of CsPb_2_Br_5_ binding on the (G) (100), (H) (110), and (I) (111) facets of Cs_0.2_FA_0.8_Pb(I_0.6_Br_0.4_)_3_. (J) Formation energy diagram before and after CsPb_2_Br_5_ binding on different Cs_0.2_FA_0.8_Pb(I_0.6_Br_0.4_)_3_ facets. (K) Gibbs free energy for the nucleation of a Cs_0.2_FA_0.8_Pb(I_0.6_Br_0.4_)_3_ crystal without and with CsPb_2_Br_5_.

Two-dimensional CsPb_2_Br_5_ has a ternary halogen-plumbate crystal structure (Fig. 1A) [40], which possesses low solubility, high stability, and Br-rich features, which has been previously used to reduce the nonradiative energy transfer and boost ionic conductivity of CsPbBr_3_ [41–43] and control the phase transition of 2-step MAPbI_3_ [44] and one-step FAPbI_3_ [45]. Therefore, it is expected that CsPb_2_Br_5_ could be potentially effective in regulating the crystallization of WBG Cs_0.2_FA_0.8_Pb(I_0.6_Br_0.4_)_3_. We synthesized CsPb_2_Br_5_ by a cooling precipitation method following the literature [45], and white powder with the shape of flakes (Fig. 1B) and typical x-ray diffraction (XRD) peaks of tetragonal structure CsPb_2_Br_5_ (Fig. 1C, PDF no. 54-0753) can be produced. High-resolution transmission electron microscopy (HRTEM) (Fig. 1D) of the CsPb_2_Br_5_ powder shows lattice spacings of 0.27 and 0.31 nm (Fig. S1), corresponding to the (210) and (005) facets of CsPb_2_Br_5_, respectively (Fig. S2). Energy-dispersive x-ray element mapping of the crystal shows a matched elemental ratio of Cs:Pb:Br ≈ 1:2:5 (Fig. S3). In addition, the selected area electron diffraction pattern extracted from HRTEM (inset in Fig. 1D) shows single-crystal diffraction spots, which can be indexed to the [001] zone axis of tetragonal CsPb_2_Br_5_ [42]. This synthesized CsPb_2_Br_5_ was further used as an additive for the deposition of Cs_0.2_FA_0.8_Pb(I_0.6_Br_0.4_)_3_ perovskite film by the antisolvent-dropping spin-coating method, and the key roles of CsPb_2_Br_5_ played during the process were comprehensively studied.

The colloidal property of the perovskite precursor solution plays a foundational role in solution-processed perovskite film [46], and the influence of CsPb_2_Br_5_ on the colloidal size and crystallization behavior of Cs_0.2_FA_0.8_Pb(I_0.6_Br_0.4_)_3_ was studied first. Dynamic light scattering measurement showed that after introducing CsPb_2_Br_5_, the average colloidal size in the precursor solution dramatically decreases from 5,518 to 825 nm, suggesting that the presence of CsPb_2_Br_5_ can interact with Cs_0.2_FA_0.8_Pb(I_0.6_Br_0.4_)_3_ precursors and reconstruct them into smaller clusters. Optical microscopy was used to monitor the crystal behavior in the precursor solutions by heating the solution. It can be seen that the control solution shows a slow nucleation rate and an unbalanced growth of nuclei with different sizes, while the target solution exhibits a fast and homogeneous nucleation together with a balanced and uniform nucleus growth (Fig. 1F). This is a typical heterocrystallization process [47,48], which indicates that the interaction between CsPb_2_Br_5_ and Cs_0.2_FA_0.8_Pb(I_0.6_Br_0.4_)_3_ can promote the nucleation and homogenize the nucleus growth of Cs_0.2_FA_0.8_Pb(I_0.6_Br_0.4_)_3_. To verify this, calculations based on density functional theory were carried out to demonstrate the influence of CsPb_2_Br_5_ on the formation energy of the Cs_0.2_FA_0.8_Pb(I_0.6_Br_0.4_)_3_ structure. The heterostructures of CsPb_2_Br_5_ binding on different facets of Cs_0.2_FA_0.8_Pb(I_0.6_Br_0.4_)_3_ were constructed (Fig. 1G to I), and the formation energies of all the 3 heterostructures were found to be lowered after binding CsPb_2_Br_5_ (Fig. 1J and Table S1). This result suggests the reduced nucleation energy of Cs_0.2_FA_0.8_Pb(I_0.6_Br_0.4_)_3_ in the presence of CsPb_2_Br_5_ (Fig. 1K), which explains the facilitated nucleation observed in the target solution. Note that Cs_0.2_FA_0.8_Pb(I_0.6_Br_0.4_)_3_ with an exposed (100) facet possesses the lowest formation energy and smallest lattice mismatch with CsPb_2_Br_5_, indicating the preferred (100) growth of Cs_0.2_FA_0.8_Pb(I_0.6_Br_0.4_)_3_ on CsPb_2_Br_5_.

**Fig. 2.**
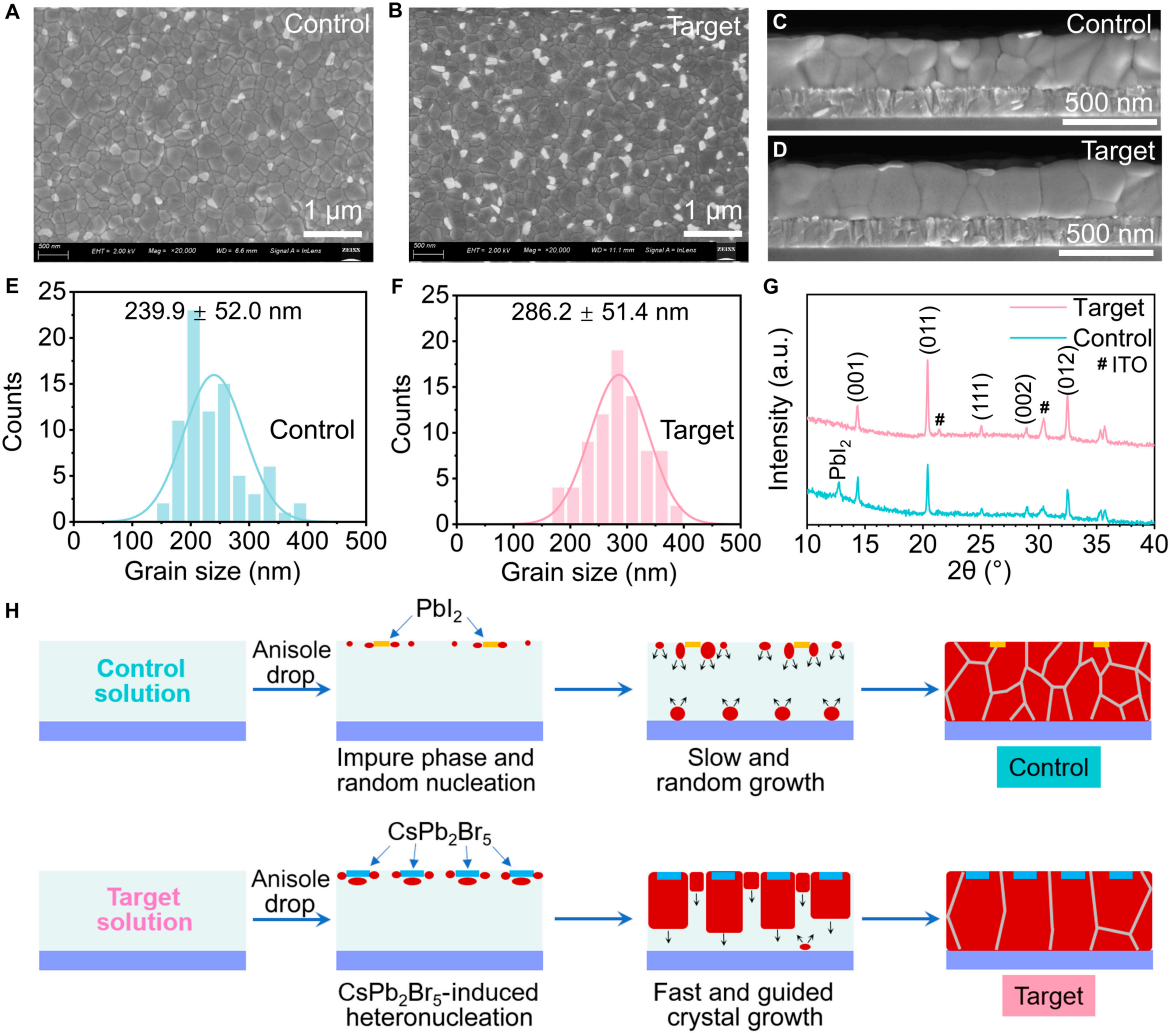
SEM images of the (A) control and (B) target Cs_0.2_FA_0.8_Pb(I_0.6_Br_0.4_)_3_ films. Cross-sectional SEM of the (C) control and (D) target films. Statistics of grain size in the (E) control and (F) target films. (G) XRD pattern of the films. (H) Illustration of the crystallization process of the films. ITO, indium tin oxide.

The microscopic morphology and crystal formation mechanism of the films were studied. From surface scanning electron microscopy (SEM), it can be seen that the control film shows a morphology of compactly arranged grains with some white particles on the surface (Fig. 2A), while the target film shows the morphology of similar compact grains but with more and brighter white flakes on the surface (Fig. 2B). Cross-sectional SEM images of the films demonstrate that the thicknesses of the both films are ~300 nm, and the control film shows vertically stacked grains with lateral grain boundaries (GBs) (Fig. 2C), while the target film is composed of vertically thorough and laterally tightly arranged crystals with much less lateral GBs (Fig. 2D). By statistical analysis of the size of the grains, the grain size is found to be enlarged from 239.9 ± 52.0 nm (Fig. 2E) to 286.2 ± 51.4 nm (Fig. 2F) after introducing CsPb_2_Br_5_. In addition, the root-mean-square roughness of the control film is 32.2 nm, which is reduced down to 25.2 nm in the target film (Fig. S4). The XRD patterns of the films show typical peaks of the Cs_0.2_FA_0.8_Pb(I_0.6_Br_0.4_)_3_ perovskite phase (Fig. 2G) [49]. Note that the control film possesses a diffraction peak at ~12° that is assigned to the PbI_2_ phase, suggesting that the white particles in the control film should be the participating PbI_2_ phase, which is typically observed in pristine Cs_0.2_FA_0.8_Pb(I_0.6_Br_0.4_)_3_ films [50]. Meanwhile, the target film shows no such PbI_2_ peak together with enhanced intensity of other perovskite-phase peaks, indicating the suppressed PbI_2_ formation and better crystallinity of the film. The bright white sheets on the surface of the target film should be the participating CsPb_2_Br_5_, which is due to its lower solubility that emerges right after antisolvent dropping. However, no XRD peak of CsPb_2_Br_5_ can be observed in the target film, which should be due to its low content. Based on the experimental evidence, we propose the following mechanism: due to its low solubility, CsPb_2_Br_5_ exists as colloidal suspended clusters within the precursor solution. Upon antisolvent dropping, these CsPb_2_Br_5_ clusters precipitate instantly at the top surface of the wet film, and they act as heterogeneous nucleation sites to template a “top-down”-guided growth of the perovskite phase. In this process, CsPb_2_Br_5_ acts as a solid seeding agent rather than undergoing a full dissolution–reprecipitation cycle, which results in the presence of residual flakes on the final film surface.

Furthermore, we depict the crystal growth process without and with CsPb_2_Br_5_ (Fig. 2H). For the control solution without CsPb_2_Br_5_, the PbI_2_ phase forms after anisole dropping due to the relatively low solubility of PbI_2_ compared to that of other precursors, and the nucleation of perovskite is slow and random. Due to the slow nucleus growth, the nucleation on the substrate starts in the meantime, resulting in the morphology of vertically stacked small grains in the control film. Meanwhile, for the target solution with CsPb_2_Br_5_, the CsPb_2_Br_5_ phase emerges at the top surface of the wet film right after anisole dropping, resulting in an ordered “top-down” crystal growth and the morphology of vertically thorough crystals with an enlarged size. This lays the foundation for the efficient vertical transportation of photogenerated charge carriers in the PSCs.

**Fig. 3.**
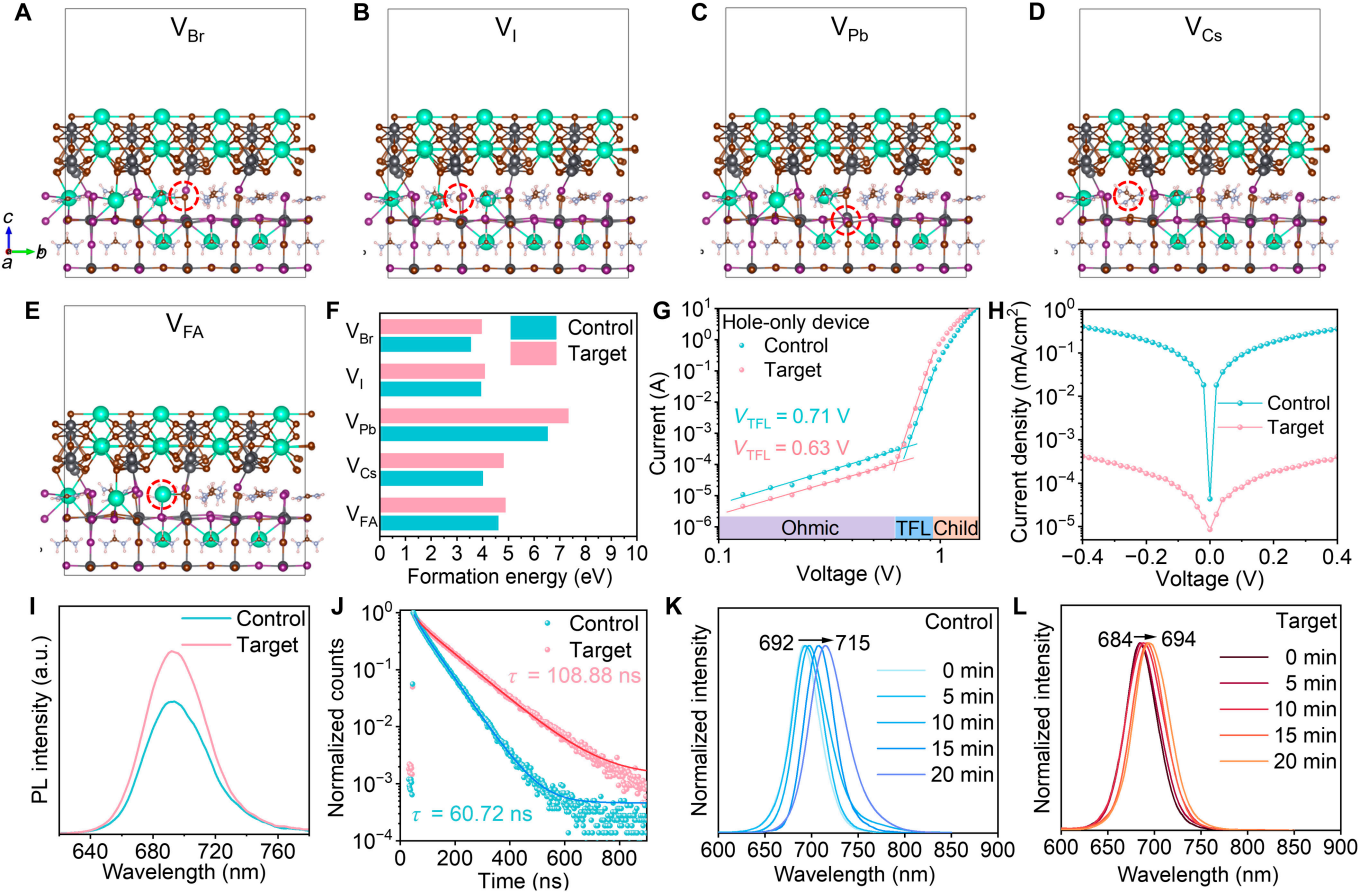
Optimized crystal structures of CsPb_2_Br_5_ binding on the (100) facet of Cs_0.2_FA_0.8_Pb(I_0.6_Br_0.4_)_3_ with vacancy defects of (A) V_Br_, (B) V_I_, (C) V_Pb_, (D) V_Cs_, and (E) V_FA_. (F) Calculated formation energy of different defects in Cs_0.2_FA_0.8_Pb(I_0.6_Br_0.4_)_3_ without and with CsPb_2_Br_5_. (G) Space-charge-limited current‌, (H) dark *J*–*V*, (I) photoluminescence (PL), and (J) time-resolved fluorescence of control and target Cs_0.2_FA_0.8_Pb(I_0.6_Br_0.4_)_3_ films. PL evaluation of (K) control and (L) target Cs_0.2_FA_0.8_Pb(I_0.6_Br_0.4_)_3_ films. TFL, trap-filling limit.

The impact of CsPb_2_Br_5_ on the defect property of Cs_0.2_FA_0.8_Pb(I_0.6_Br_0.4_)_3_ was then investigated. Density functional theory calculations were adopted to show the change in the formation energies of 5 vacancy defects (i.e., V_Br_, V_I_, V_Pb_, V_Cs_, and V_FA_) before and after introducing CsPb_2_Br_5_ (Fig. 3A to E). Considering that the lowest formation energy of Cs_0.2_FA_0.8_Pb(I_0.6_Br_0.4_)_3_ is based on the heterostructure of CsPb_2_Br_5_ binding on the exposed (100) facet (Fig. 1G), the defects were created based on this structure. Note that although the XRD of the final film showed a higher proportion of the (110) facet (Fig. 2G), the initial nucleation could occur on the (100) facet due to the lowest formation energy and smallest lattice mismatch. It is shown that the formation energy of all these vacancy defects increases after introducing CsPb_2_Br_5_ (Fig. 3F and Table S2), suggesting a reduced defect density in the formed crystal, which should be beneficial for inhibiting the defect-mediated phase separation in Cs_0.2_FA_0.8_Pb(I_0.6_Br_0.4_)_3_ [51–53]. The space-charge-limited current‌ of the hole-only device was measured to characterize the trap-state density in the deposited Cs_0.2_FA_0.8_Pb(I_0.6_Br_0.4_)_3_ film, and the control and target films showed trap-filling limit voltages (*V*_TFL_) of 0.71 and 0.63 V, respectively (Fig. 3G), which corresponds to a reduced trap-state density from 1.66 × 10^16^ cm^−3^ (control) to 1.47 × 10^16^ cm^−3^ (target). In addition, by measuring the dark *J*–*V* curve of PSCs (Fig. 3H), the target device showed a much decreased dark current, which agrees with the reduced defect density in the film. Moreover, the target film exhibited an increased photoluminescence (PL) intensity (Fig. 3I) with a significantly prolonged PL lifetime from 60.72 to 108.88 ns (Fig. 3J), demonstrating the much suppressed nonradiative recombination in the film owing to the reduced trap-state density. Note that the position of the PL peak in the target film showed a blueshift from 694 to 690 nm, which is consistent with the ultraviolet–visible spectra of the films that showed a blueshifted absorption edge, and the corresponding Tauc plots demonstrate an enlarged bandgap from 1.79 to 1.80 eV (Fig. S5). The photoinduced phase separation feature of the films was tested by monitoring their PL peak variation under continuous AM1.5 illumination, and the target film exhibited a retarded peak shift compared to the control one after continuous illumination for 20 min (Fig. 3K and L), implying suppressed photoinduced phase separation owing to the reduced defect density that inhibits the defect-mediated halide migration in the CsPb_2_Br_5_-regulated film.

**Fig. 4.**
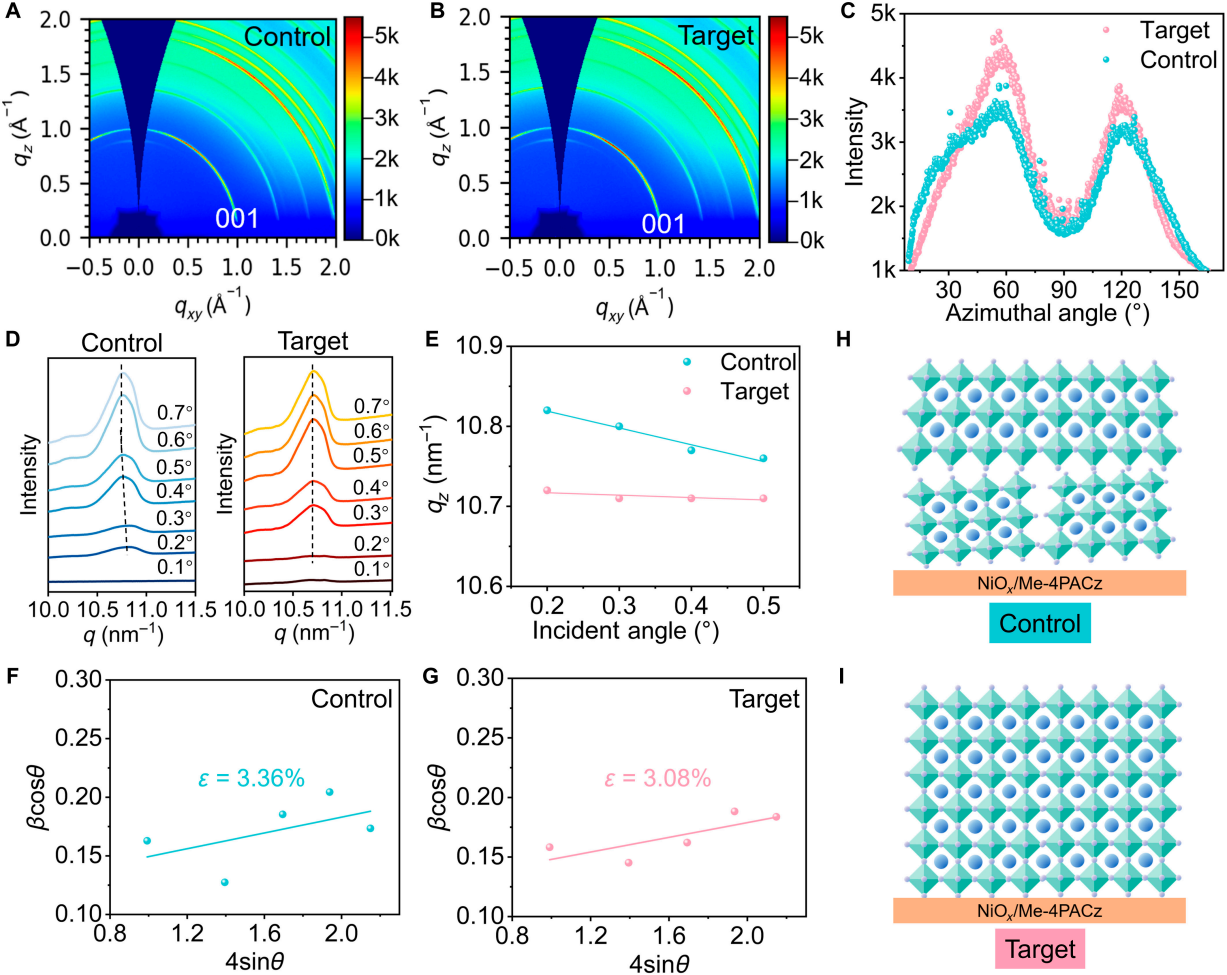
Grazing incidence wide-angle x-ray scattering (GIWAXS) of the (A) control and (B) target Cs_0.2_FA_0.8_Pb(I_0.6_Br_0.4_)_3_ films. (C) Radially integrated intensity plots as a function of azimuthal angle along the ring at *q_z_* ≈ 10 nm^−1^ of the films. (D) Incident-angle-dependent GIWAXS profiles of the films. (E) Peak position at *q_z_* ≈ 10 nm^−1^ in the function of the incident angle of the GIWAXS of the films. (F) XRD patterns and (G) calculated lattice strains based on Williamson–Hall plots extracted from the XRD patterns of the films. Schematics of the strain conditions in the (H) control and (I) target films. Me-4PACz, ([6-(4-methylphenyl)-9*H*-carbazole-3,6-bis(diphenylphosphine oxide).

The crystallographic properties of the prepared films were then studied. The grazing incidence wide-angle x-ray scattering (GIWAXS) profiles of the films were measured to reflect the crystal orientation of the films by analyzing the Debye–Scherrer rings of (001) facets at *q* ≈ 1.0 Å^−1^. Both the films showed a Bragg spot emerging in the direction of ~60° on the (001) ring (Fig. 4A and B), corresponding to the included angle of ~60° between the (001) crystal plane and the substrate. The intensity plots of the (100) ring of the 2 films were azimuthally integrated to provide detailed orientation information (Fig. 4C), and it was observed that the target film exhibited an increased intensity and a narrowed peak width at ~60° and ~120°, suggesting the more preferred orientation after introducing CsPb_2_Br_5_. Notably, previous work has also observed this unique orientation of Cs_0.2_FA_0.8_Pb(I_0.6_Br_0.4_)_3_ film [49], which could be the advantageous crystal orientation of Cs_0.2_FA_0.8_Pb(I_0.6_Br_0.4_)_3_ for charge transport. Therefore, this CsPb_2_Br_5_-induced enhanced orientation is beneficial for charge transport. The residual strains along the vertical direction of the films were then characterized by incident-angle-dependent GIWAXS via probing the position change of the typical diffraction peak at different film depths (Figs. S6 to S8). The peak of the (001) facet at ~*q* = 10.7 nm^−1^ was selected to demonstrate the vertical strain distribution (Fig. 4D), and considering that an incident angle of 0.4° corresponds to a probe depth of ~400 nm [54,55], an incident angle in the range of 0.1° to 0.5° is sufficient to reflect the strains in the perovskite films (Fig. 4E). It can be seen that the control film shows a peak shift toward a small *q* value as the probe depth increases, while a negligible peak shift can be observed in the target film, indicating the much reduced tensile strains along the vertical direction of the film enabled by the CsPb_2_Br_5_-regulated growth of the film. Williamson–Hall plots were extracted from the XRD patterns of the films as presented in Fig. 4F and G and Figs. S9 and S10, and the residual strain *ε* of the films can be estimated by the slope of the linear fitting of the Williamson–Hall plots (see the Supplementary Materials for details). It is calculated that the residual strain in the film is reduced from 3.36% to 3.08% in the presence of CsPb_2_Br_5_, suggesting the released strain enabled by CsPb_2_Br_5_-guided growth of perovskite in the target film (Fig. 4H and I).

**Fig. 5.**
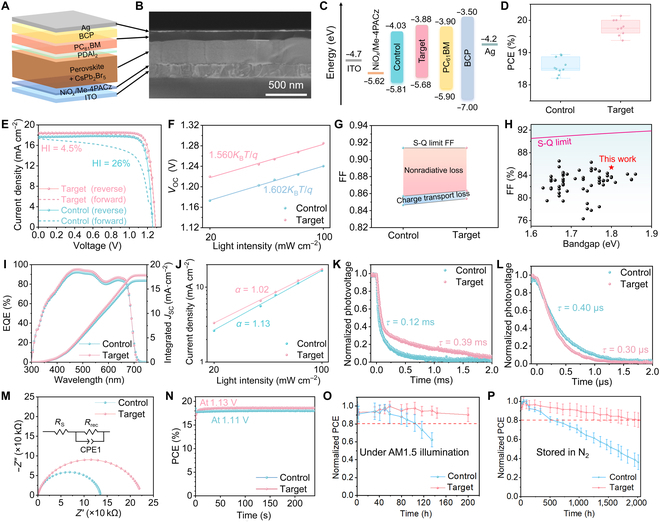
(A) Device structure, (B) cross-sectional SEM, and (C) energy level diagram of perovskite solar cells (PSCs). BCP, 2,9-dimethyl-4,7-diphenyl-1,10-phenanthroline. (D) Power conversion efficiency (PCE) statistics of 10 individual devices, (E) *J*–*V* curves under both scan directions, (F) light-intensity-dependent *V*_OC_, and (G) fill factor (FF) loss mechanism with respect to the Shockley–Queisser (S-Q) limit of the control and target devices. HI, hysteresis index. (H) Comparison of bandgap-dependent FF values reported in the literature and in this work. (I) External quantum efficiency (EQE), (J) light-intensity-dependent *J*_SC_, (K) normalized transient photovoltage decay, (L) normalized transient photocurrent decay, (M) electrochemical impedance spectroscopy (EIS), and (N) maximum power point (MPP) tracking of the control and target devices. PCE variations of unencapsulated devices tested under the conditions of (O) 1.5 AM illumination and (P) storage in N_2_.

Further, the photovoltaic performance of the films was studied by constructing p–i–n PSCs (Fig. 5A). The cross-sectional SEM image of the target device shows a typical planar structure, where the perovskite layer is composed of vertically thorough and tightly arranged crystals (Fig. 5B). The energy levels of the 2 films were characterized by ultraviolet photoelectron spectroscopy (Figs. S11 and S12) together with bandgaps obtained from Tauc plots, and the resultant energy level diagram of the PSC device is depicted in Fig. 5C. It is observed that the target film shows an upshifted valence band maximum from −5.81 to −5.68 eV and an upshifted conduction band minimum from −4.03 to −3.88 eV, both of which match more with the energy levels of the respective charge transport layers, suggesting a better charge extraction in the target device. Note that the Kelvin probe force microscopy measurement of the target film shows an increased surface potential compared to that of the control one (Fig. S13), which agrees with the upshifted energy levels. The PCE distributions of 10 individual PSCs were collected (Fig. S14), and the target devices exhibited improved photovoltaic parameters with a statistical PCE of 19.77% ± 0.23% compared to the control ones with 18.58% ± 0.26% (Fig. 5D). Notably, the target device exhibits a champion PCE of 20.14% with a *V*_OC_ of 1.285 V, a *J*_SC_ of 18.36 mA cm^−2^, and an FF of 85.39%, which are superior to those of the control device with a PCE of 18.99%, a *V*_OC_ of 1.25 V, a *J*_SC_ of 17.93 mA cm^−2^, and an FF of 84.65%, and the hysteresis index of target device was also significantly reduced from 26% to 4.5% (Fig. 5E and Table S3). To the best of our knowledge, a PCE of 20.14% is the highest value for formamidinium (FA)/Cs-based 1.80-eV PSCs so far (Table S4 and Fig. S15). By measuring the light intensity-dependent *V*_OC_ (Fig. 5F), the FF loss mechanism was analyzed to gain a deeper understanding of the origin of the FF improvement. There are 2 FF loss pathways for real PSCs to be unable to reach the S-Q limit FF, including nonradiative loss and charge transport loss. Furthermore, considering the condition without charge transport loss, the maximum FF (FF_max_) in this case can be estimated [56], which is calculated to be 85.70% and 86.35% for the control and target PSCs, respectively (see the Supplementary Materials for calculation details). By comparing FF_max_ with the FF we measured in the devices (Fig. 5G), it is found that both the trap-assisted nonradiative loss and the charge transport loss are suppressed in the target device, which is assigned to the reduced defect density, reduced lateral GBs, enhanced orientation, released strains, and more matched energy levels in the target device. Notably, an FF value of 85.39% is also the record-high value so far for FA/Cs-based 1.80-eV PSCs (Table S3 and Fig. 5H). External quantum efficiency (EQE) measurement demonstrates that the target device has a higher photoelectric conversion response in the range from 400 to 670 nm (Fig. 5I), and the integrated *J*_SC_ values are 16.72 and 17.83 mA cm^−2^, respectively, which are both in agreement with the *J*_SC_ values obtained from the *J*–*V* curves. Moreover, light-dependent *J*_SC_ variation shows that the target device presents a reduced *α* value from 1.13 to 1.02 (Fig. 5J), demonstrating much suppressed bimolecular recombination in the target device. In addition, the transient photovoltage decay tracking under open-circuit conditions of the target device shows an increased decay time from 0.12 to 0.39 ms (Fig. 5K), while the transient photocurrent decay tracking under short-circuit conditions of the target device shows a decreased decay time from 0.4 to 0.3 μs (Fig. 5L), and these results can be also attributed to the less nonradiative recombination in the device. Electrochemical impedance spectroscopy was further carried out to verify the efficient charge transport in the device, and the Nyquist plots of the target device shows an increased charge recombination resistance (*R*_ct_) from 135,760 to 224,760 Ω compared to that of the control one (Fig. 5M), again demonstrating suppressed nonradiative recombination. Photocurrent tracking under the maximum power point was measured under AM1.5 illumination conditions, and the target device showed stabilized power output at 1.13 V (Fig. 5N). The illumination stability of the unencapsulated devices was tested under light soaking conditions, and the time for the target device to maintain 80% of its initial PCE is much prolonged compared to that of the control one (Fig. 5O). Under storage conditions, the target device retains ~80% of its original PCE after storage in N_2_ for 2,000 h, while the PCE of the control device dropped to less than 80% of its initial value after only 300 h (Fig. 5P). In addition, the target devices also show enhanced thermal (heating at 60 °C) and air stability (stored under 30 °C room temperature and 70% relative humidity) compared to the control ones (Fig. S16).

**Fig. 6.**
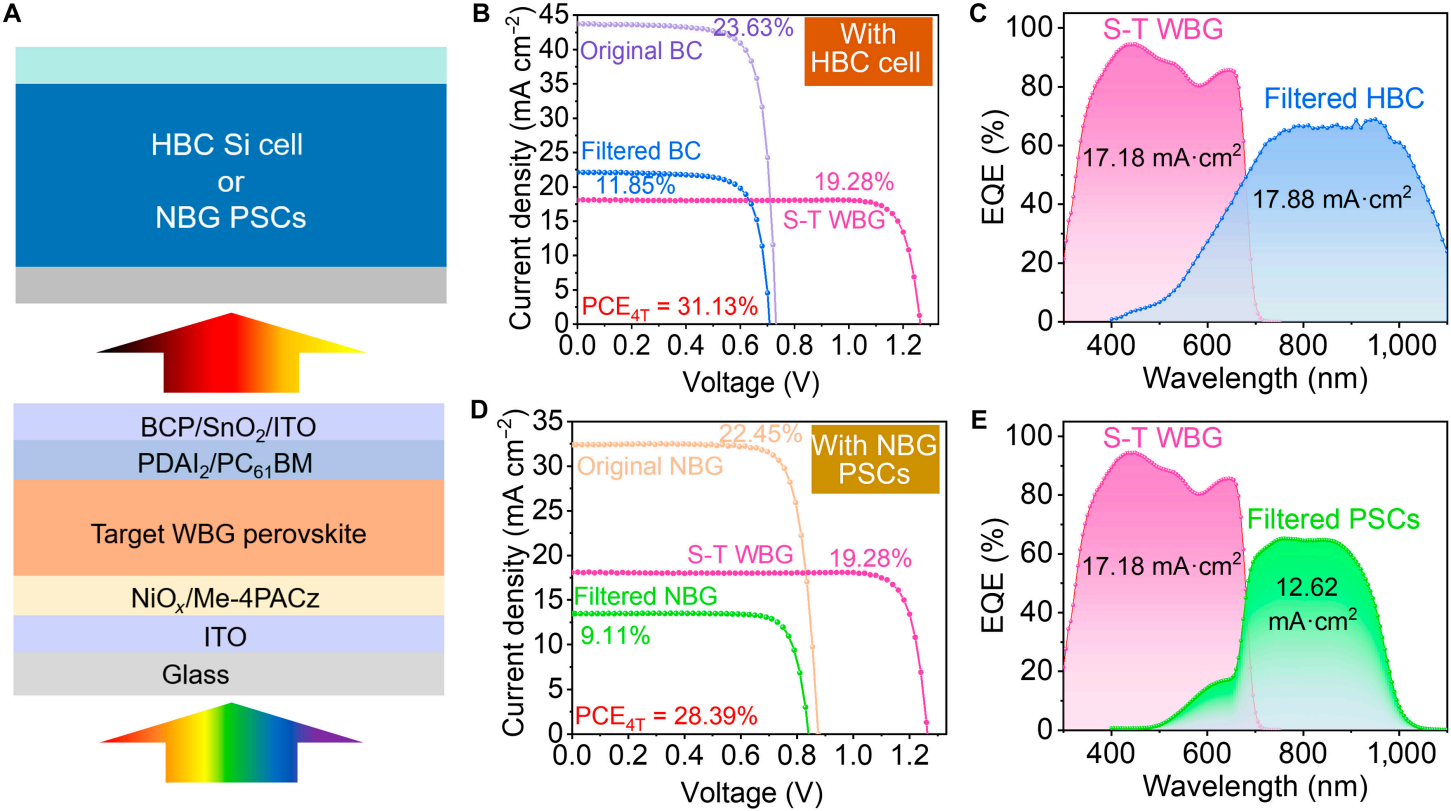
(A) Illustration of the structure of the 4-terminal (4T) tandem device using a heterojunction back contact (HBC) Si cell or narrow-bandgap (NBG) PSCs as the top subcell. (B) *J*–*V* curves and (C) EQE of perovskite/silicon tandem solar cells. (D) *J*–*V* curves and (E) EQE of all-perovskite tandem solar cells.

Finally, the potential application of the 1.80-eV WBG PSCs in tandem photovoltaic devices was demonstrated, and 4T tandem devices were constructed (Fig. 6A), where an HBC Si cell or NBG PSCs (FA_0.7_MA_0.3_Pb_0.5_Sn_0.5_I_3_) serve as the top subcell and an S-T WBG device with a PCE of 19.28% (Fig. S17 provides the transmission spectra of the S-T WBG device) serves as the bottom subcell. The HBC silicon cell with a PCE of 23.63% produced a filtered PCE of 11.85%, resulting in the 4T perovskite/silicon tandem device yielding a PCE of 31.13% (Fig. 6B) with a stabilized power output of 30.81% (Fig. S18), which is superior to that of the tandem device based on the control WBG PSCs (Fig. S19). The EQEs of the S-T WBG and filtered HBC silicon device are shown in Fig. 6C, from which the *J*_SC_ values are integrated to be 17.18 and 17.88 mA cm^−2^, respectively. Moreover, the PCE statistics of the 4T tandem device based on the target WBG devices show a higher performance compared to those of the control ones (Fig. S20). We also fabricated 1-cm^2^ S-T WBG PSCs with a PCE of 16.67% to construct a 4T tandem device, yielding a PCE of 28.33% (Fig. S21). Moreover, an all-perovskite tandem device was also constructed, which achieved a decent PCE of 28.39% (Fig. 6D) with a stabilized power output of 28.01% (Fig. S22). Moreover, the EQEs of the filtered NBG device (Fig. 6E) produced an integrated *J*_SC_ of 12.62 mA cm^−2^, which agrees with the filtered *J*–*V* curve of the NBG device. All these results demonstrate the great promise of the WBG PSCs we developed in the application of perovskite-based tandem photovoltaics.

## Conclusion

In conclusion, we have developed an effective crystallization regulator, 2-dimensional CsPb_2_Br_5_, for Cs_0.2_FA_0.8_Pb(I_0.6_Br_0.4_)_3_ WBG perovskite to realize efficient 1.80-eV WBG PSCs and their perovskite/silicon tandem device. Experimental and theoretical studies showed that CsPb_2_Br_5_ can guide the vertical crystallization and suppress the defect formation of the WBG film, which resulted in the efficient charge transport, released residual strains, and inhibited phase separation of the WBG perovskite film. After optimization, the 1.80-eV WBG PSCs achieved record-high PCE of 20.14% and FF of 85.39% together with enhanced irradiation and storage stability. By combining with an HBC silicon NBG subcell, the 4T tandem devices yielded promising PCEs of 31.13% and 28.39% in perovskite/silicon and all-perovskite tandems, respectively. This work highlights the critical role of rational crystallization control in boosting the performance of WBG PSCs and their tandem devices.

## Materials and Methods

Information about the materials and methods used in this work is available in the Supplementary Materials.

## Data Availability

All data are available from the corresponding authors on reasonable request.

## Supplementary Materials

Supplementary 1 Experimental Section Figs. S1 to S22 Tables S1 to S4
