# Supplementary material for: Heterointeraction-Induced Nucleation Promoting Vertical Growth and Suppressing Phase Separation for Efficient Wide-Bandgap Perovskite Solar Cells and Tandem Devices
Source: Research (Wash D C). 2025 Sep 22;8:0892. doi: 10.34133/research.0892 (PMC12451108; doi:10.34133/research.0892)
Supplement: Supplementary 1 — Experimental Section Figs. S1 to S22 Tables S1 to S4 [file research.0892.f1.zip › 20250814 SI-WBG PSCs.docx]

**Supporting Information**

**Hetero-nucleation promoting vertical growth and suppressing phase separation for efficient wide-bandgap perovskite solar cells and tandem devices**

Changbo Li,^1+^ Yuyi Wang,^1+^ Weiyin Gao,^1,3+*^ Jianxiong Yang,^1^ Zelin Wang,^1^ Xiaojia Zhao,^1^ Xinyuan Liu,^1^ Liangxu Wang,^1^ Yile Liu,^1^ Xiaobo Wang,^1^ He Dong,^1^ Long Zhou,^2^ Weidong Zhu,^2*^ Chenxin Ran^1,4,5*^, Wei Huang^1*^

^1^Frontiers Science Center for Flexible Electronics, Xi’an Institute of Flexible Electronics (IFE) and Northwestern Polytechnical University, Xi'an 710072, China.

^2^State Key Laboratory of Wide-Bandgap Semiconductor Devices and Integrated Technology, Xidian University, Xi’an, 710071, P.R. China

^3^Engineering Research Center of Smart Energy and Carbon Neutral in Oil & Gas Field Universities of Shaanxi Province, College of New Energy, Xi’an Shiyou University, Xi’an 710065, China.

^4^Research & Development Institute of Northwestern Polytechnical University in Shenzhen, Shenzhen 518063, China.

^5^Chongqing Innovation Center, Northwestern Polytechnical University, Chongqing 401135, China.

+These authors contributed equally to this work.

E-mail: [iamwygao@xsyu.edu.cn](mailto:iamwygao@xsyu.edu.cn); [wdzhu@xidian.edu.cn](mailto:wdzhu@xidian.edu.cn); [iamcxran@nwpu.edu.cn](mailto:iamcxran@nwpu.edu.cn); [vc@nwpu.edu.cn](mailto:vc@nwpu.edu.cn)

**Experimental Section**

**Materials**

Chlorobenzene (CB, 99.8%), Dimethyl sulfoxide (DMSO, 99.97%), N-dimethylformamide (DMF, 99.8%), lead bromide (PbBr_2_, 99.999%), Anisole (An, 99.7%) were purchased from Sigma-Aldrich. Lead iodide (PbI_2_, 99.99%)，[4-(3,6dimethyl-9H-carbazol-9-yl)butyl]phosphonic acid (Me-4PACz, 99%), dibutylammonium iodide (PDAI_2_, 98%) were purchased from TCI. Formamidinium iodide (FAI, 99.9%), cesium iodide (CsI, 99.9%), cesium monobromide (CsBr, 99.9%), NiO_X_ nanoparticle powder, PC_61_BM were purchased from Advanced Election Technology. Ethyl Alcohol (ETOH, 99.7% AR), isopropanol (IPA, 99.7% AR) were purchased from Sinopharm Chemical Reagent Co., Ltd. BCP (99.5%) were purchased from Jinlin OLED Material Tech Co., Ltd.

**Synthesis of CsPb_2_Br_5_ nanocrystals**

42.6 mg (0.2 mmoL) CsBr was dissolved in 2 ml H_2_O and stirred until completely dissolved. Then, 146.8 mg PbBr_2_ (0.4 mmoL) was dissolved in 4 ml HBr solution and stirred until completely dissolved. After this, 2 mL PbBr_2_ solution was gradually added to the 4 mL CsBr solution, and then CsPb_2_Br_5_ white precipitate was produced. The product was wash by ethanol for three times, and then centrifuged at 3000 rpm for 3 min. The poweder was finally dry in a vacuum oven for 12 hours to obtain the white product.

**Fabrication of 1.80eV perovskite films and devices**

Preparation of perovskite precursor solution. 165.1 mg FAI (0.96 mmoL), 62.4 mg CsI (0.24 mmoL), 221.3 mg PbI_2_ (0.48 mmoL), 264.5 mg PbBr_2_ (0.72 mmoL) and 22 mg CsPb_2_Br_5_ (0.023 mmoL)were dissolved in 200 µL of DMSO and 800 µL of DMF, stirred 2 h at room temperature to prepare a 1.2 M FA_0.8_Cs_0.2_Pb(I_0.6_Br_0.4_)_3_ precursors solution, and filtered through a 0.22 μm PTFE filter membrane for use.

Preparation of Me-4PACz precursor solution. 0.6 mg Me-4PACz were dissolved in a 1 mL ETOH to prepare a 0.6 mg/mL solution, stirred thoroughly at room temperature, and then filtered through a 0.22 μm PTFE filter membrane for use.

Preparation of NiO_X_ precursor solution. 10 mg NiO_X_ were dissolved in a 1 mL mixed solution of IPA and H_2_O (1:3 v/v) to make a 10mg/mL solution, after dispersing with ultrasound for 10 min, which was filtered through a 0.22 μm PTFE filter membrane for use.

Preparation of PDAI_2_ precursor solution. 0.5 mg PDAI_2_ were dissolved in a 1 mL IPA to prepare a 0.5 mg/mL solution, stirred thoroughly at room temperature, and then filtered through a 0.22 μm PTFE filter membrane for use.

Preparation of PC_61_BM precursor solution. 18 mg PC_61_BM were dissolved in a 1 mL CB to prepare a 18 mg/mL solution, stirred thoroughly at room temperature, and then filtered through a 0.22 μm PTFE filter membrane for use.

Deposition of HTL layer. The ITO glass was treated by UV-ozone for 20 min. Firstly, NiO_X_ was spin-coated on ITO at 3000 rpm for 25 s, and annealed at 100 °C for 10 min. After that, Me-4PACz was spin-coated on ITO at 3000 rpm for 25 s, and annealed at 100 °C for 10 min.

Fabrication of perovskite film. 50 µL perovskite precursor solution was spin-coated on substrates 5000 rpm 32s, and 100 µL of antisolvent (An) was rapidly dropped at 24 s followed by annealed at 100 °C for 10 min. After cooling to room temperature, 38 µL PDAI_2_ solution (0.5 mg/mL) was spin-coated at 3000 rpm for 25 s and then annealed at 100 °C for 5 min.

Fabrication of device. After cooling the films to room temperature, PCBM was spin-coated at 1000 rpm 50s and 3000 rpm for 3 seconds. The device is completed by thermal evaporation of 6 nm BCP and 100 nm Ag electrode at the rate of 0.2 Å/s and 2 Å/s. Semi-transparent WBG perovskite films and device is prepared by the following methods. The device was fabricated by depositing a 20 nm layer of tin oxide (SnO_x_) using atomic layer deposition (ALD) technology, and a 100 nm layer of indium tin oxide (ITO) was sputtered at a power of 50 W.

**Precursor solution and film characterization**

The crystallization of the precursor solution on the substrates was observed by RTX series biological microscope and the static images were captured. Field Emission Scanning Electron Microscope (SEM) images were captured using the ZEISS Gemini SEM 300 (Carl Zeiss AG) at an accelerating voltage of 3 kV. Time-of-Flight Secondary Ion Mass Spectrometry (ToF-SIMS) of the perovskite film was performed and analyzed by Nanjing Demo Science Limited laboratory using a 3KV, 100 nA Ar ion cluster to sputter a 600×600 µm^2^ sample surface with a cycle period of 10 s. In-situ UV-vis absorption spectra were obtained on a multi-spectrometer (DU-200, Shaanxi Puguang Weishi Co.Ltd.). X-ray diffraction (XRD) patterns were obtained using the Bruker D8 diffractometer with a maximum tube voltage of 60 kV and maximum tube current of 80 mA. Grazing Incidence Wide Angle X-Ray Scattering (GIWAXS) patterns were performed at the BL14B1 beamline of the Shanghai Synchrotron Radiation Facility (SSRF), using a wavelength of 1.24 Å, detector distance from sample of 359.45 mm, and grazing incidence angles of 0.1-0.6°. Atomic force microscopy (AFM) images were taken with the Cypher S Asylum Research Cypher S. (UV-Vis) were acquired using the Hitachi UH5700 absorption spectrophotometer. Kelvin Probe Force Microscopy (KPFM) images were taken using the Bruker Dimension Icon. UV-Visible spectrophotometer (UV-Vis) were acquired using the Hitachi UH5700 absorption spectrophotometer. Photoluminescence spectrum (PL) were obtained using the Edinburgh Instruments FLS1000 steady-state-transient fluorescence spectrometer. Ultraviolet Photoelectron Spectroscopy (UPS) were taken using the Shimadzu Kratos Axis Supra.

**Device characterization**

All the devices were tested in an N_2_-filled glovebox using a Keithley 2400 source meter under a simulated AM1.5G spectrum and a solar simulator (EnliTech, Taiwan). The light intensity was calibrated to be 100 mW cm^-2^ using a NREL-certified monocrystalline Si diode (Newport 532 ISO1599). Data acquisition was carried out using a K-2400 source meter with a scanning range of -0.2 to 1.35 V, with a scanning step of 0.02 V/step and a dwell time of 1 ms. The device performance parameters were calculated from the current-voltage curves of the photovoltaic device under 100 mW cm^-2^ with shadow masks area of 0.05 cm^2^. Maximum power point (MPP) voltage was record for 180s under continuous standard AM 1.5G illumination. Dark current tests are conducted in the absence of external light stimulation, using a K-2400 digital source meter for recording and analysis. Electrochemical Impedance Spectroscopy (EIS) were recorded on Chenhua CH760 E. External Quantum Efficiency (EQE) was tested using the QTEST HIFINITY 5 external quantum efficiency measurement system under room temperature in air. Stability testing was performed at room temperature in a glove box filled with N_2_.

For the Space Charge Limited Current (SCLC) test, the defect density of states is obtained from the equation:

*N*_trap_ = 2*ε*_0_*εV*_TFL_/*qL*^2^

where *N*_trap_ is the density of states of the defect, *V*_TFL_ is the initial bias voltage when the defect is filled, *ε*_0_ is vacuum permittivity (8.82 × 10^-14^ F/cm^2^), *ε*_r_ is relative permittivity of perovskite (~34), *q* is the amount of charge carried by a unit of positive charge (1.6 × 10^-19^ C), and *L* is the thickness of the light-absorbing layer of the perovskite film. The lattice strain ɛ of the films can be extracted from XRD patterns by fitting the slope in the Williamson-Hall plot:

bcosq = 4esinq+Kl/D_size_

where, ɛ is the lattice strain, β is the half width of the characteristic peak of XRD, θ is the Bragg angle in radians, l is the X-ray incident wavelength, K is the sharp factor, D_size_ is crystallite size.

The FF loss between the Shockley-Queisser limit and the measured FF is caused by non-radiative loss and charge transfer. Transmission loss composition, according to the formula:

$$\text{F}\text{F}_{\text{max}}\text{ = }\frac{\text{υ}_{\text{OC}}\text{-}\ln\text{(}\text{υ}_{\text{OC}}\text{+}\text{0.72}\text{)}}{\text{υ}_{\text{OC}}\text{+}\text{1}}$$

$$\text{υ}_{\text{OC}}\text{ = }\frac{\text{V}_{\text{OC}}}{\text{n}\text{k}_{\text{B}}\text{T}\text{/}\text{q}}$$

where, $\text{q}$ is the basic charge, $\text{T}$ is the thermodynamic temperature at room temperature, $\text{k}_{\text{B}}$ is the Boltzmann constant, and $\text{V}_{\text{OC}}$ is the open circuit voltage of the device. The maximum *FF* (*FF_max_*) that can be achieved without charge transfer loss can be calculated from the formula.

The HI was calculated according to the equation as follows:

$$\text{HI}\text{ }\text{= }\frac{\text{PCE}_{\text{RS}}\text{-}\text{PCE}_{\text{FS}}}{\text{PCE}_{\text{RS}}}$$

Where in PCE_RS_ is the PCE obtained by reverse scanning, and PCE_FS_ is the PCE obtained by forward scanning.

**Computational details**

All the spin-polarized density functional theory calculations were conducted by using the Vienna ab initio simulation package (VASP). The exchange-functional is treated using the generalized gradient approximation (GGA) of Perdew-Burke-Ernzerhof (PBE) functional. The D3 correction approach (DFT-D3) was employed in order to reflect the impact of van der Waals (vdW) interactions. The projector-augmented wave (PAW) approach was used to signify core-valence interactions. The energy cutoff for the plane wave basis expansion was set to 500 eV. Optimized structures were obtained by minimizing the forces on each ion using the conjugate gradient algorithm until they are below 0.02 eV/Å. The self-consistent calculations apply a convergence energy threshold of 10^-5^ eV. Brillouin zone was sampled at the Γ-point. The binding energy (E_binding_) was calculated according to the following equation:

E_binding_ = E_A+B_ - E_A_ - E_B_

where E_A+B_ is the total energy of the heterogeneous structure, and E_A_ and E_B_ are the energies of the A part and the B part, respectively. A negative E_binding_ value represents an exothermic binding, and a more negative value corresponds to a stronger binding.

The formation energy of vacancies (E_formation_) was calculated according to the following equation:

E_formation_ = E_surface-vac_ + E_vac_ - E_surface_

where E_surface-vac_ and E_surface_ are the total energies of the optimized surfaces with and without a vacancy, respectively, and E_vac_ is the energy of the vacancy atoms. A positive E_formation_ value indicates that extra energy is needed to create the vacancy.


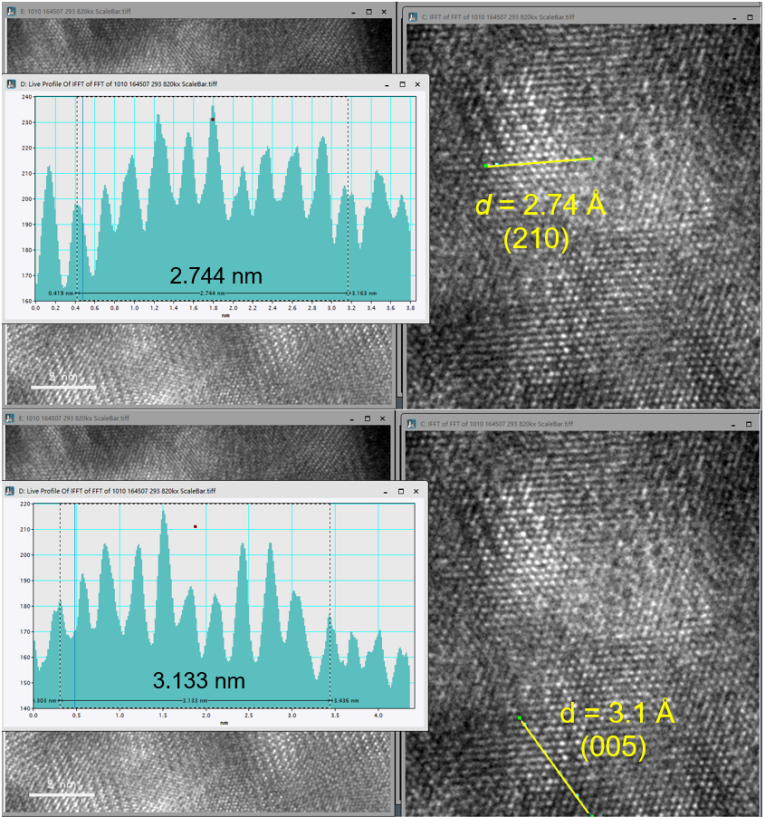


**Figure S1.** TEM images and lattice distance measurement of CsPb_2_Br_5_.


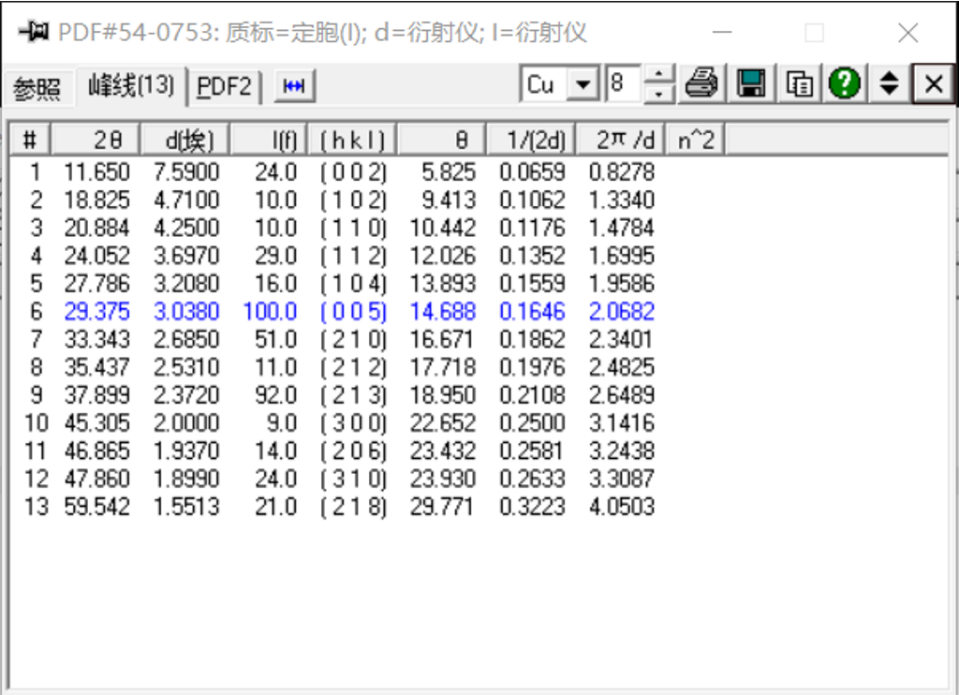


**Figure S2.** PDF card intercepts the table of different crystal plane spacing of CsPb_2_Br_5_.


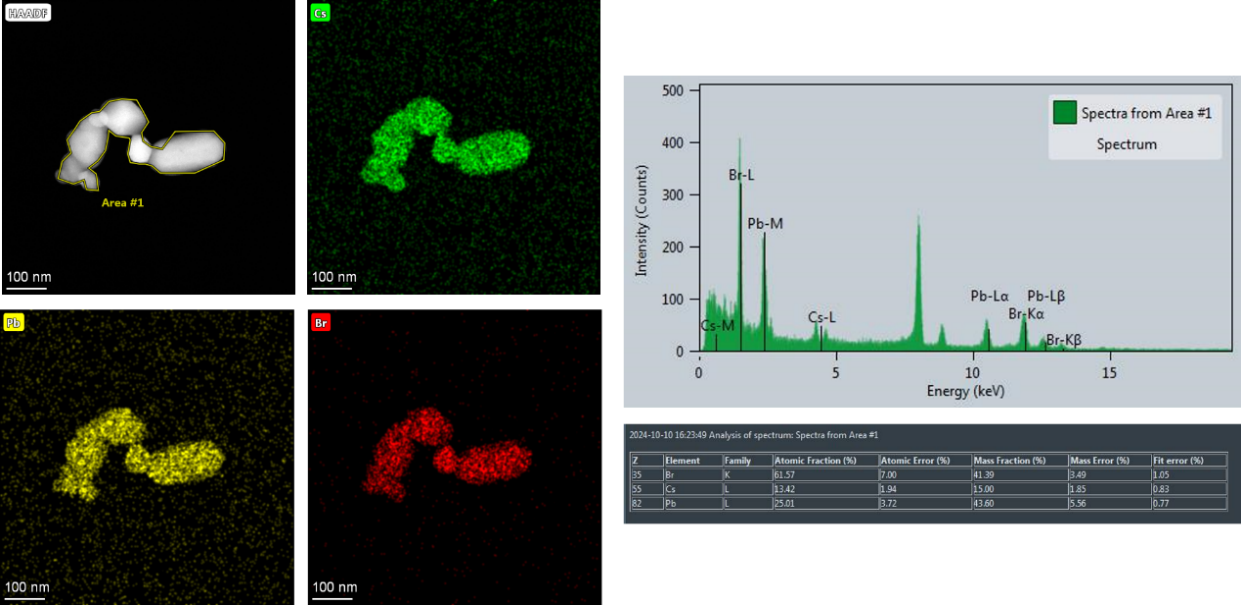
**Figure S3.** EDX mapping and contents diagram of different elements in CsPb_2_Br_5_ sample.


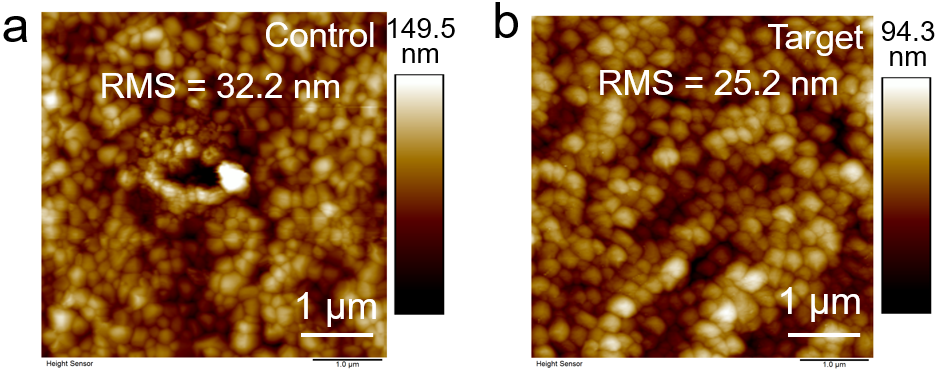


**Figure S4.** AFM images of (a) control and (b) target films.


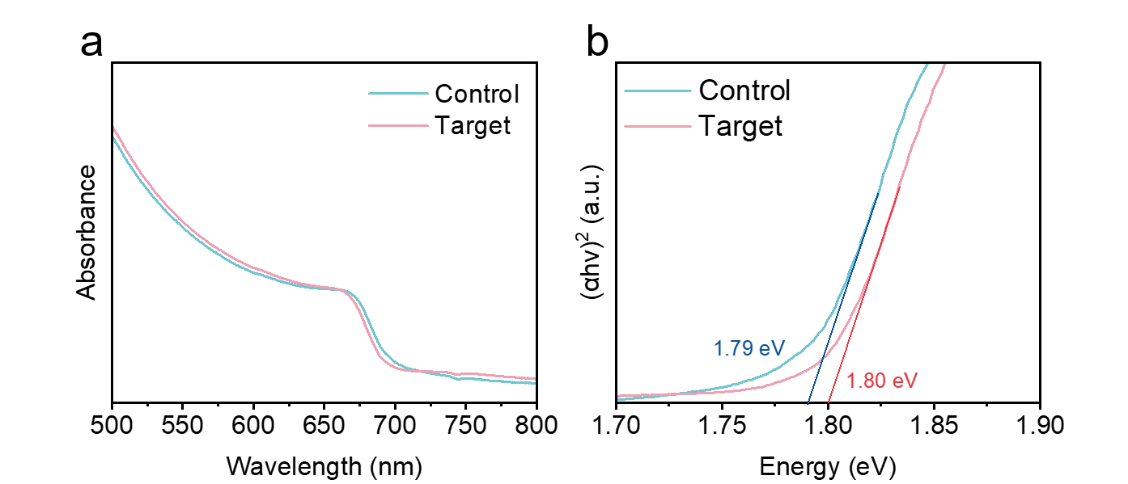


**Figure S5.** (a) UV-Vis spectra and (b) corresponding Tauc plots obtained from UV-Vis of control and target films.


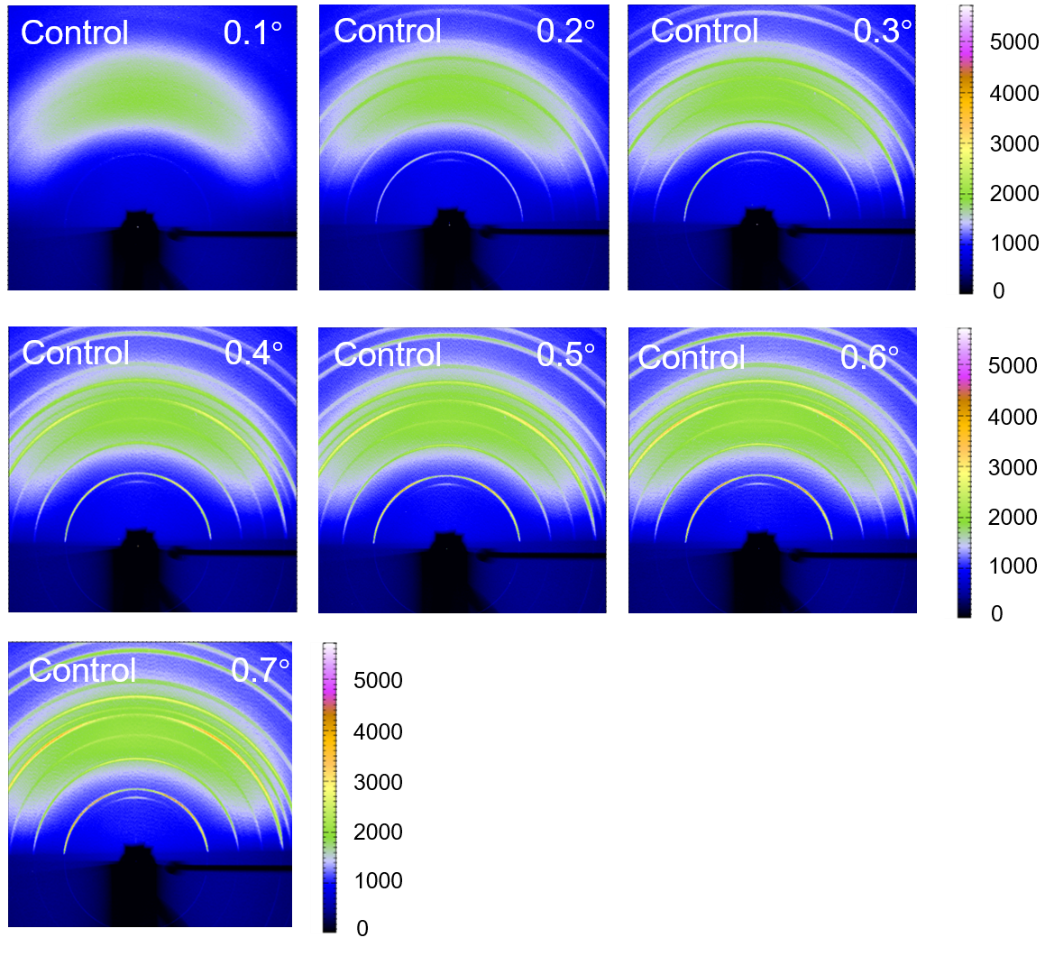


**Figure S6.** GIWAXS profiles of Control film at increased incident angles.


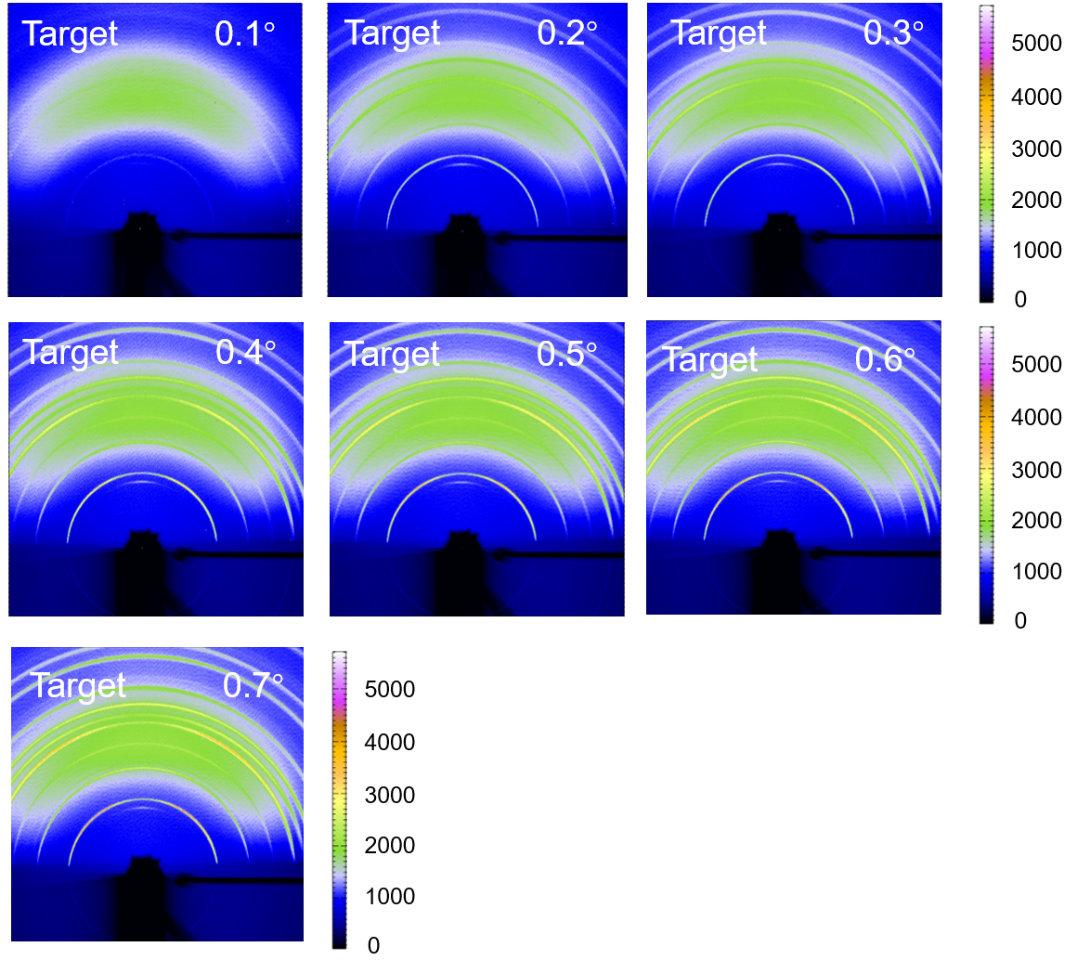


**Figure S7.** GIWAXS profiles of Target film at increased incident angles.


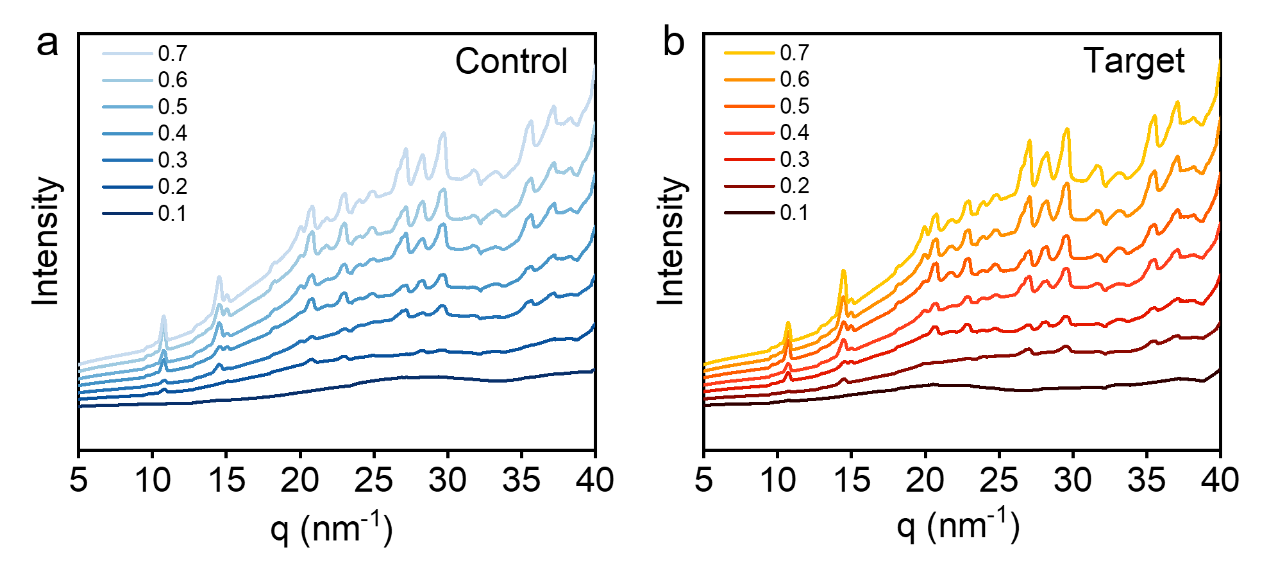


**Figure S8.** Intensity profiles obtained from depth-dependent GIWAXS of (a) Control film and (b) Target film.


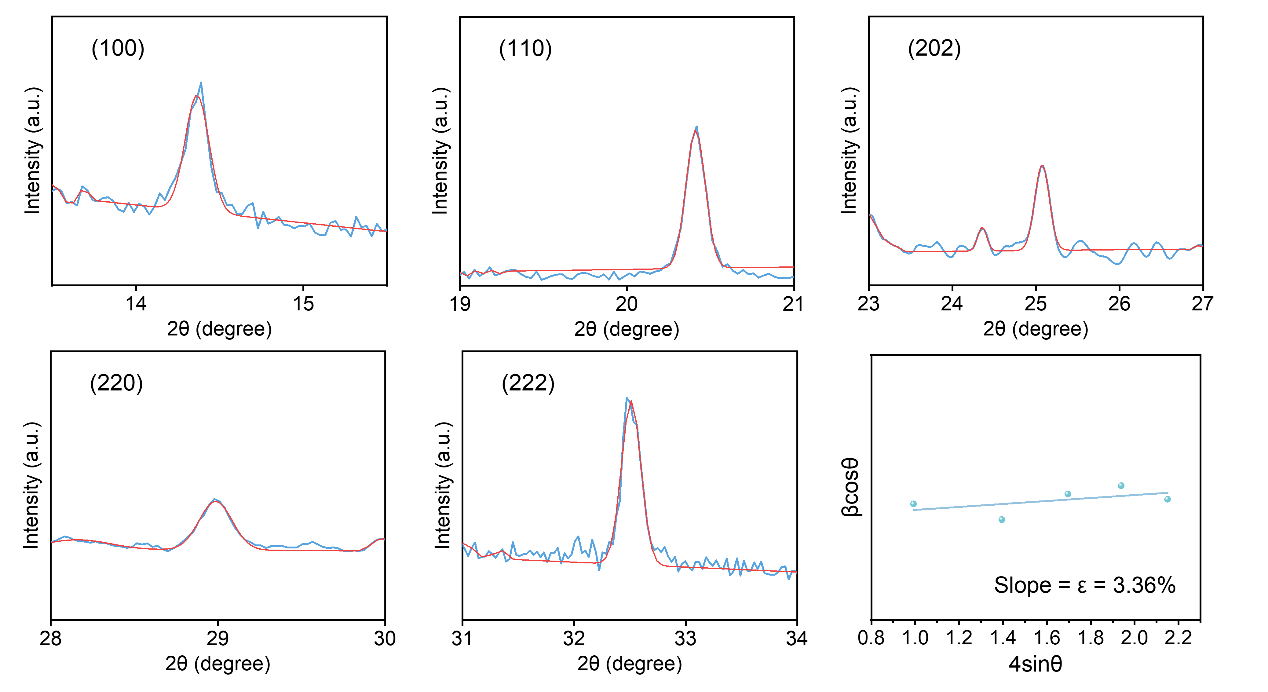
 **Figure S9.** Peak fitting of XRD patterns of Control based on Williamson-Hall analysis.


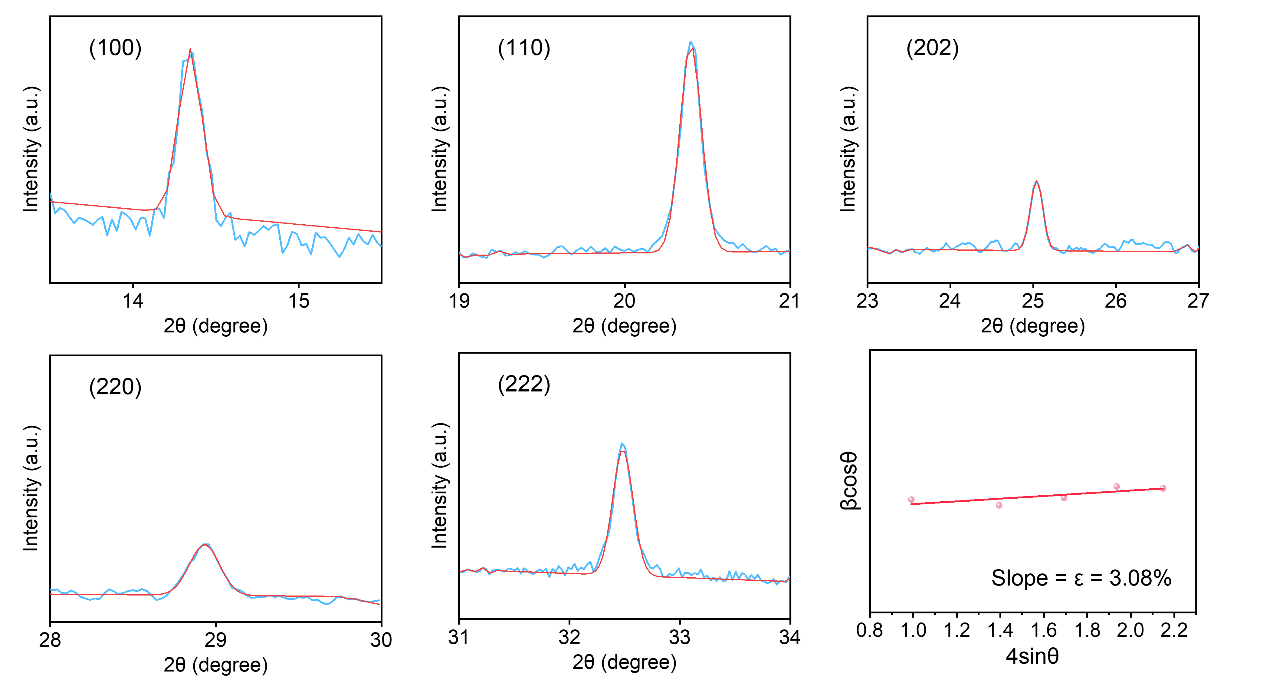
 **Figure S10.** Peak fitting of XRD patterns of Target based on Williamson-Hall analysis.


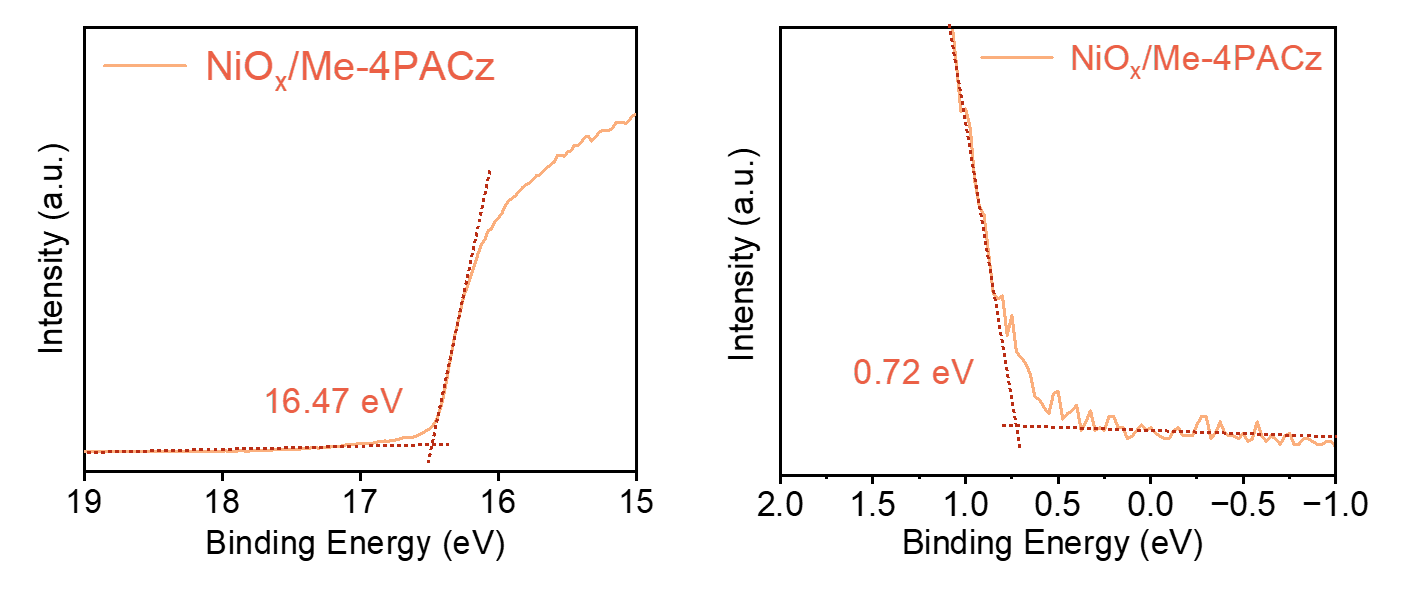


**Figure S11.** Secondary electron cut-off and valence band region obtained from UPS spectra of NiO_x_/Me-4PACz film.


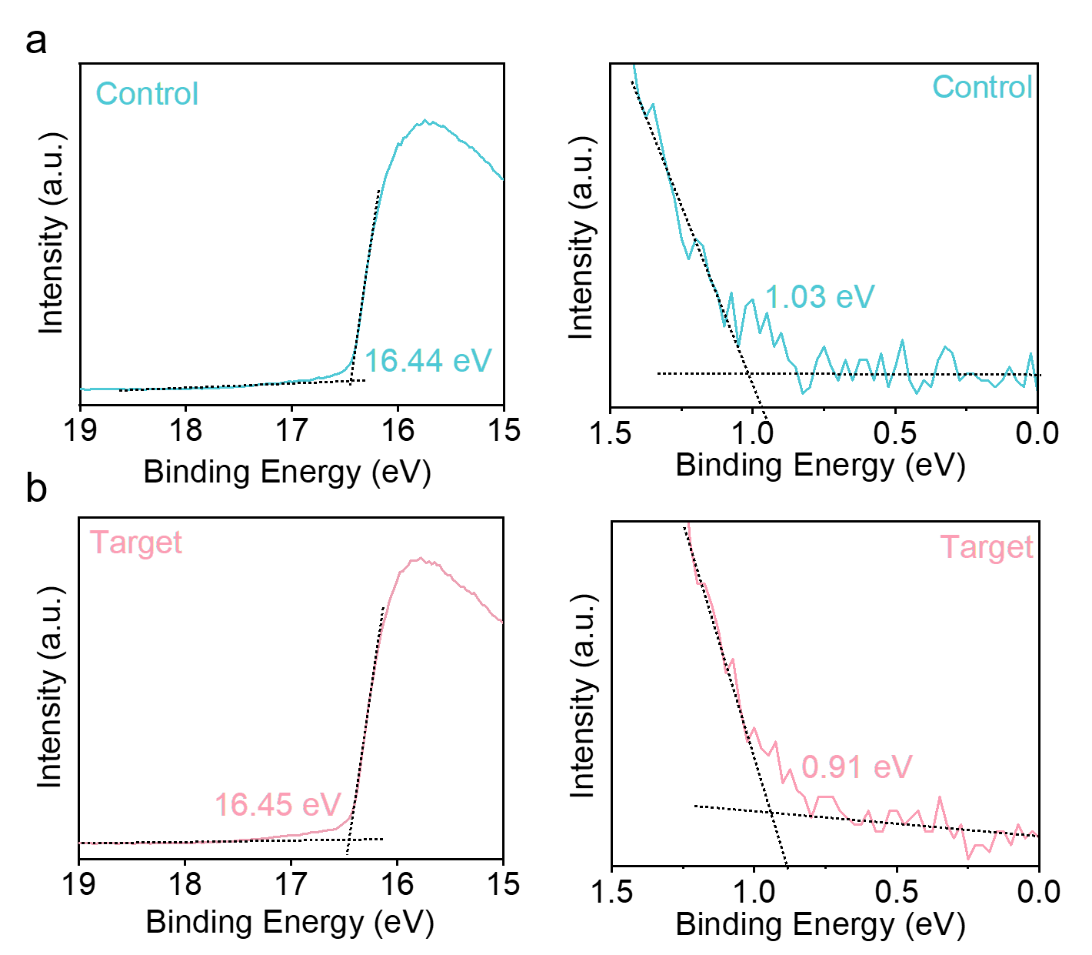


**Figure S12.** Secondary electron cut-off and valence band region obtained from UPS spectra of (a) Control film and Target film.


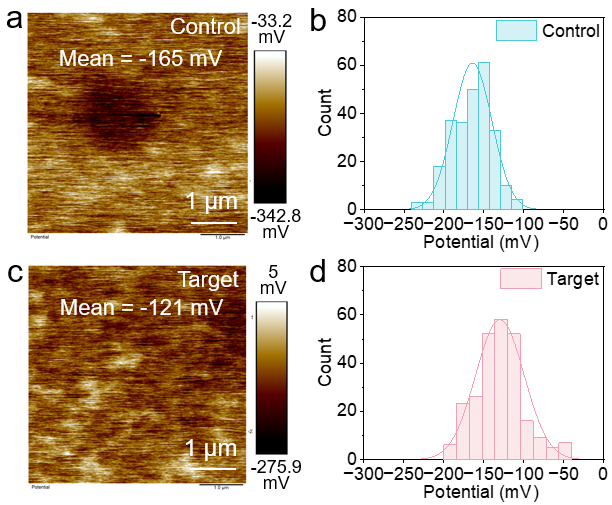


**Figure S13.** FPFM and surface potential distribution of (a, b) Control and (c, d) Target films.


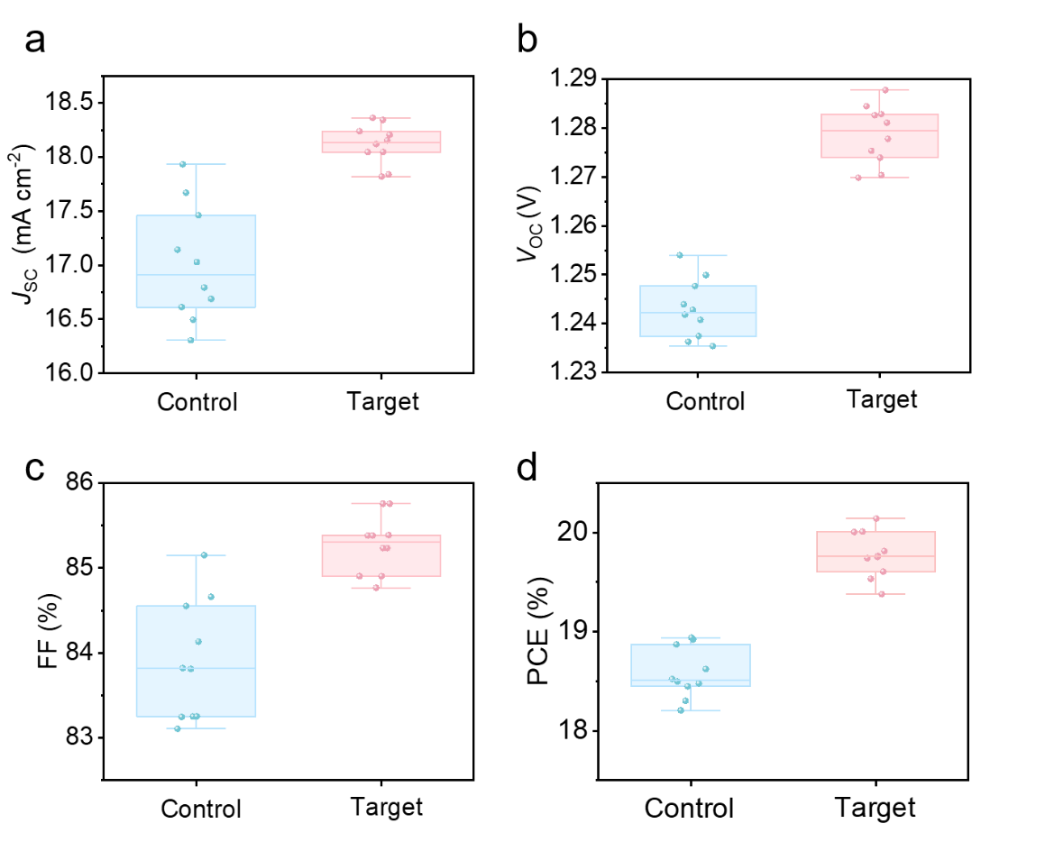


**Figure S14.** Statistics of (a) *J*_SC_ (b) *V*_OC_ (c) FF and (d) PCE distribution of the PSCs based on Control film and Target film.


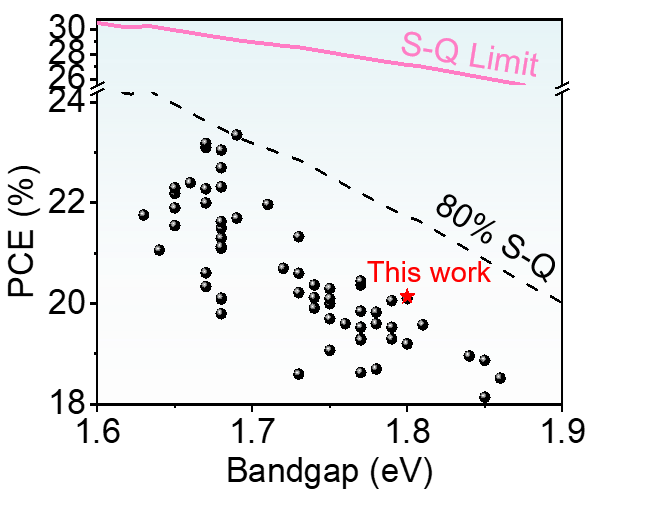


**Figure S15.** Bandgap-dependent PCEs reported in the literatures and in this work.


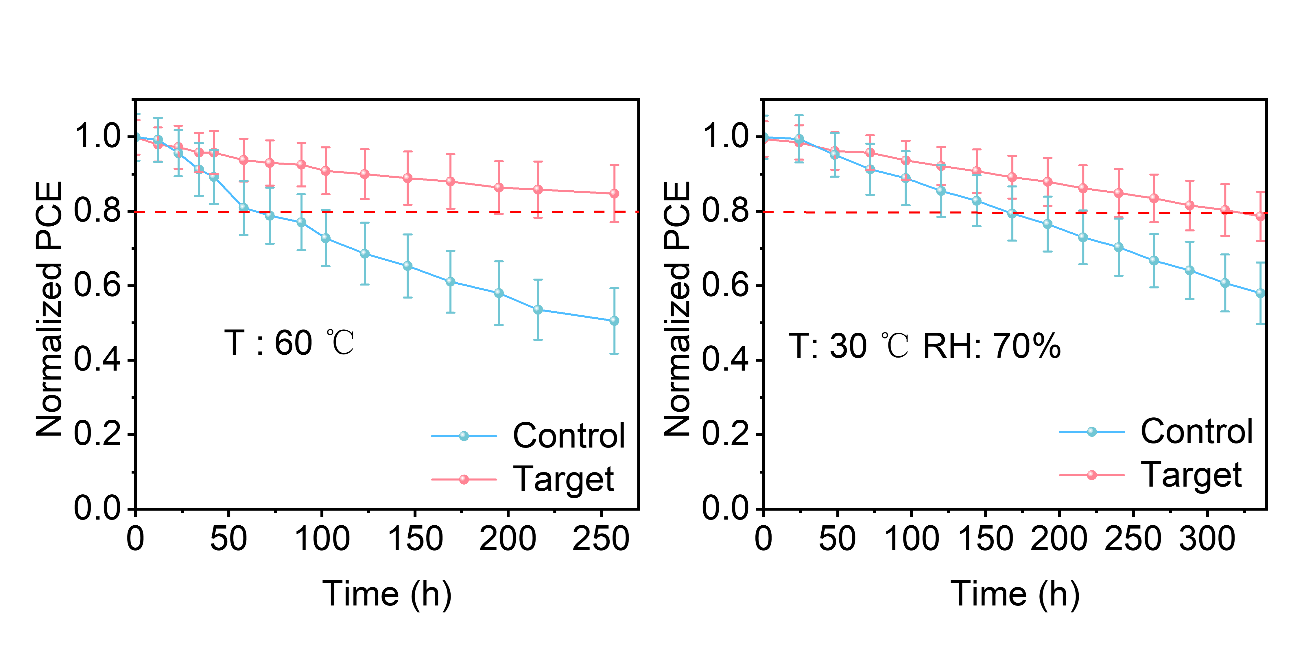


**Figure S16.** PCE variations of unencapsulated devices tested under the conditions of heating at 60°C and RH of 70% at 30°C.


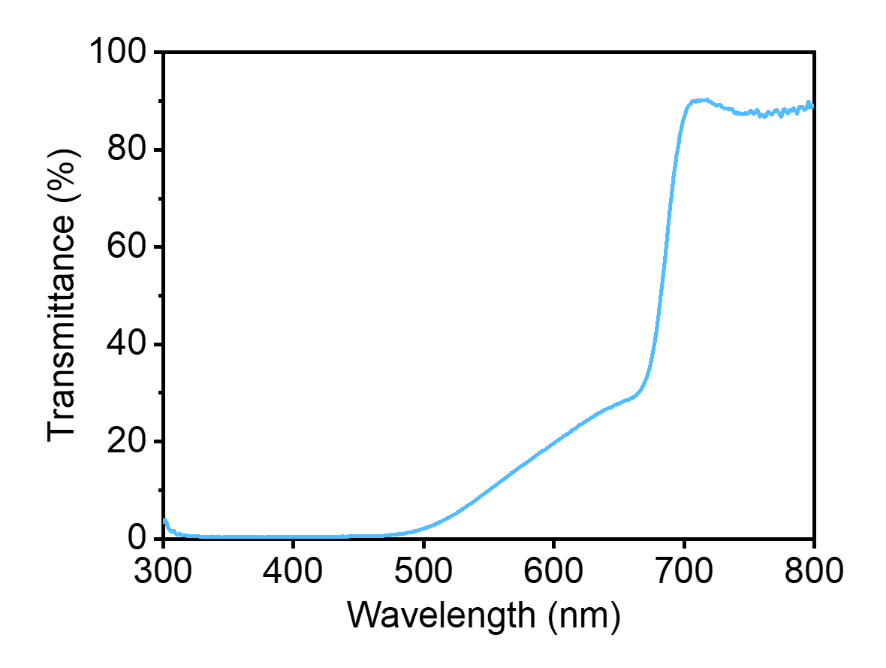


**Figure S17.** Transmittance of S-T WBG device.


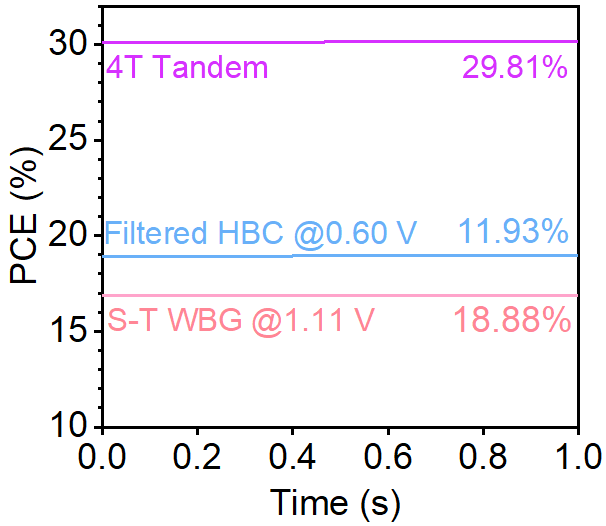


**Figure S18.** Steady-state PCE tracking at MPP of S-T WBG device and filtered HBC device.


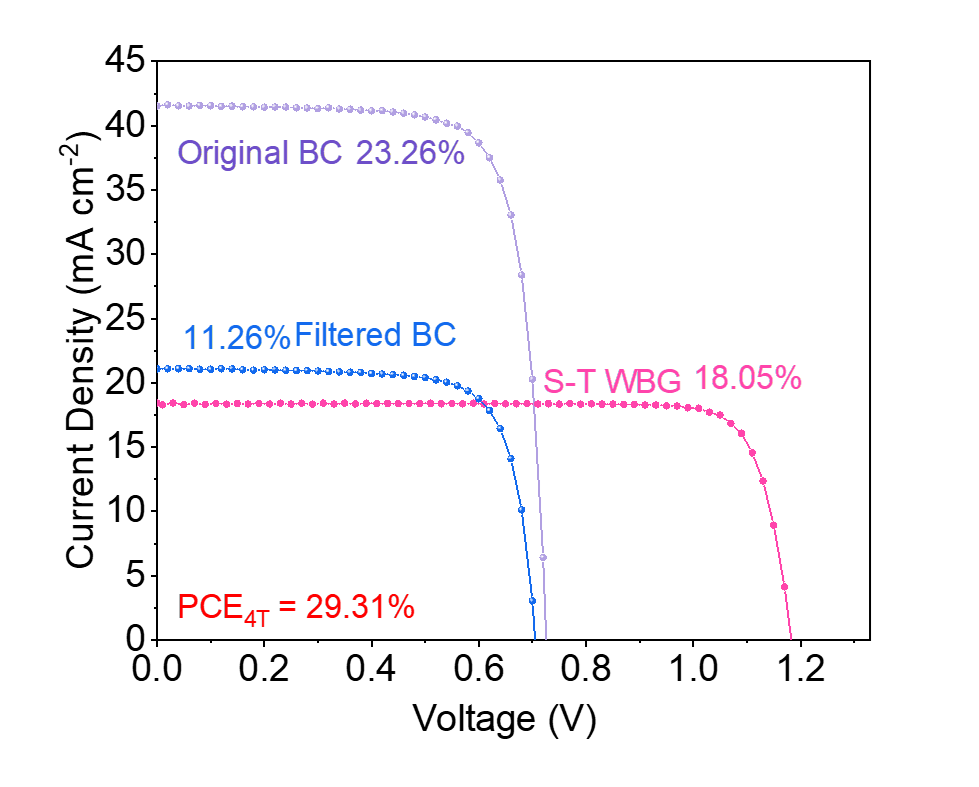


**Figure S19.** *J-V* curves of 4T perovskite/silicon tandem solar cells based on Control S-T WBG devices.


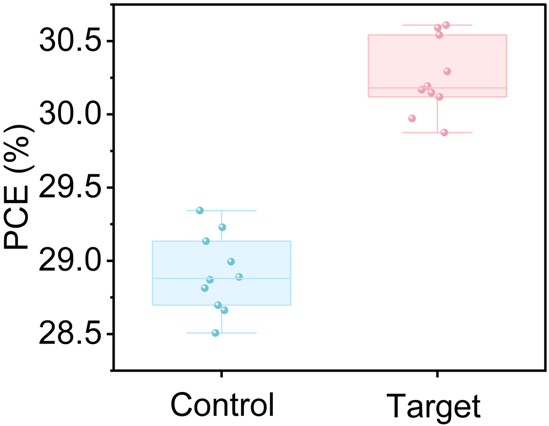


**Figure S20.** PCE statistics of the 4T tandem device based on the Control and Target WBG devices.


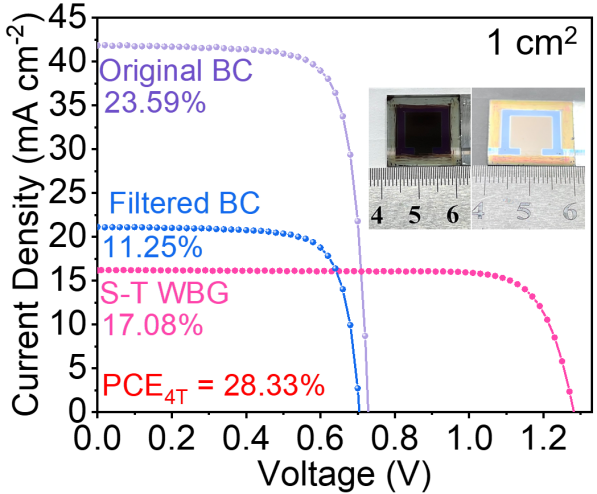


**Figure S21.** *J-V* curves of 4T perovskite/silicon tandem device on 1 cm^2^.


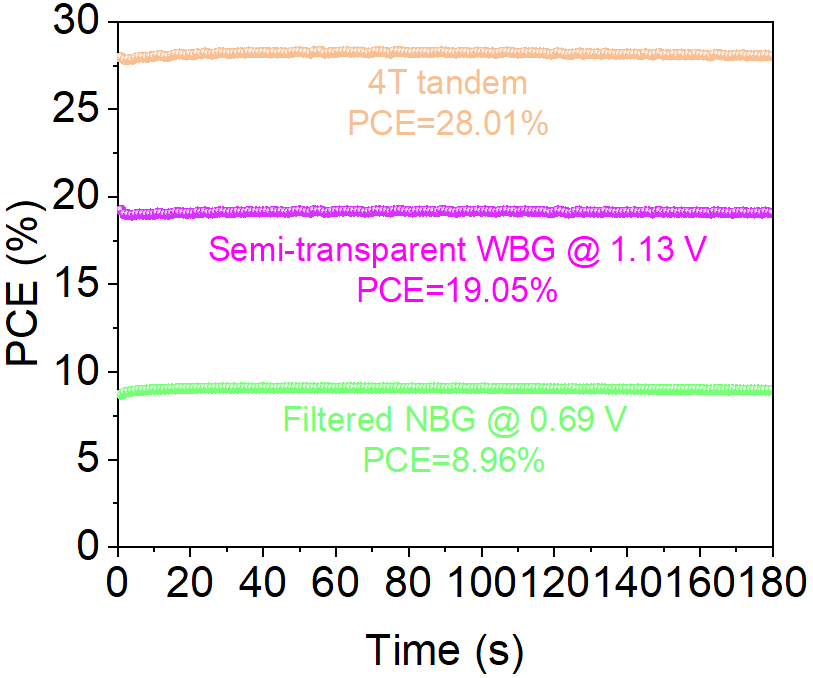


**Figure S22.** Steady-state PCE tracking at MPP of S-T WBG device and filtered NBG PSCs.

**Table S1.** Formation energy of WBG perovskite with different exposed facets without and with CsPb_2_Br_5_.

| Facets | w/o CsPb_2_Br_5_  formation energy (keV) | with CsPb_2_Br_5_  formation energy (keV) |
| --- | --- | --- |
| (100) | -1.45715 | -1.87879 |
| (110) | -0.82845 | -1.24583 |
| (111) | -1.13357 | -1.56106 |

**Table S2.** Formation energy of different vacancy defects without and with CsPb_2_Br_5_.

| Defect type | w/o CsPb_2_Br_5_  formation energy (eV) | with CsPb_2_Br_5_  formation energy (eV) |
| --- | --- | --- |
| V_Br_ | 3.5363 | 3.9552 |
| V_Cs_ | 4.0016 | 4.8062 |
| V_FA_ | 4.6096 | 4.8824 |
| V_I_ | 3.9371 | 4.0766 |
| V_Pb_ | 6.5287 | 7.3287 |

**Table S3.** Device parameters of WBG perovskite solar cell in this work.

| Additive | Scan direction | *V*_OC_  (V) | *J*_SC_ (mA/cm^2^) | FF  (%) | PCE  (%) | *HI* (%) |
| --- | --- | --- | --- | --- | --- | --- |
| Target | Reverse | 1.285 | 18.36 | 85.39 | 20.14 | 4.5 |
|  | Forward | 1.286 | 18.38 | 81.25 | 19.23 |  |
| Control | Reverse | 1.251 | 17.93 | 84.65 | 18.99 | 26 |
|  | Forward | 1.253 | 17.34 | 67.55 | 14.68 |  |

**Table S4.** Summary of the state-of-the-art WBG PSCs reported in the literatures

| Year | Band Gap  (eV) | *V*_OC_  (V) | *J*_SC_  (mA/cm^2^) | FF  (%) | PCE  (%) | Ref. |
| --- | --- | --- | --- | --- | --- | --- |
| 2024 | 1.68 | 1.24 | 20.6 | 84 | 21.5 | ^[1]^ |
|  | 1.78 | 1.31 | 18.3 | 82.3 | 19.6 |  |
| 2024 | 1.65 | 1.26 | 21 | 83 | 21.9 | ^[2]^ |
| 2024 | 1.67 | 1.26 | 20.05 | 81.57 | 20.61 | ^[3]^ |
| 2024 | 1.68 | 1.25 | 22.31 | 83 | 23.05 | ^[4]^ |
| 2024 | 1.68 | 1.19 | 21.7 | 82.3 | 21.3 | ^[5]^ |
| 2024 | 1.68 | 1.27 | 20.88 | 84.49 | 22.32 | ^[6]^ |
| 2024 | 1.69 | 1.28 | 22.43 | 81.2 | 23.35 | ^[7]^ |
| 2024 | 1.71 | 1.25 | 21.56 | 83.4 | 21.97 | ^[8]^ |
| 2024 | 1.77 | 1.29 | 18.01 | 83.08 | 19.3 | ^[9]^ |
| 2024 | 1.77 | 1.26 | 18.17 | 83.53 | 19.28 | ^[10]^ |
| 2024 | 1.77 | 1.34 | 18.16 | 83.62 | 20.37 | ^[11]^ |
| 2024 | 1.8 | 1.26 | 18.25 | 83.74 | 19.2 | ^[12]^ |
| 2024 | 1.81 | 1.35 | 17.52 | 82.74 | 19.58 | ^[13]^ |
| 2024 | 1.84 | 1.32 | 17.06 | 84.21 | 18.96 | ^[14]^ |
| 2024 | 1.86 | 1.37 | 16.1 | 84.2 | 18.52 | ^[15]^ |
| 2023 | 1.67 | 1.24 | 21.7 | 85.5 | 23.1 | ^[16]^ |
|  | 1.69 | 1.25 | 20.7 | 84.2 | 21.7 |  |
|  | 1.72 | 1.27 | 19.2 | 84.9 | 20.7 |  |
|  | 1.75 | 1.29 | 18 | 84.8 | 19.7 |  |
|  | 1.78 | 1.31 | 17.2 | 83 | 18.7 |  |
| 2023 | 1.73 | 1.3 | 19.68 | 83.27 | 21.33 | ^[17]^ |
|  | 1.79 | 1.34 | 17.8 | 83.1 | 19.53 |  |
|  | 1.85 | 1.36 | 16.21 | 83.21 | 18.14 |  |
| 2023 | 1.64 | 1.11 | 23.9 | 79.5 | 21.06 | ^[18]^ |
|  | 1.67 | 1.14 | 22.06 | 80.88 | 20.34 |  |
| 2023 | 1.67 | 1.22 | 22.67 | 80.35 | 22.28 | ^[19]^ |
|  | 1.77 | 1.19 | 20.28 | 84.75 | 20.45 |  |
| 2023 | 1.68 | 1.25 | 21.5 | 84.5 | 22.7 | ^[20]^ |
|  | 1.8 | 1.34 | 17.9 | 83.6 | 20.1 |  |
| 2023 | 1.65 | 1.24 | 21.29 | 81.77 | 21.55 | ^[21]^ |
| 2023 | 1.78 | 1.32 | 17.9 | 83 | 19.6 | ^[22]^ |
| 2023 | 1.65 | 1.26 | 21.6 | 81.6 | 22.19 | ^[23]^ |
| 2023 | 1.66 | 1.27 | 21.14 | 83.13 | 22.4 | ^[24]^ |
| 2023 | 1.67 | 1.27 | 21.09 | 82.01 | 22 | ^[25]^ |
| 2023 | 1.67 | 1.25 | 21.49 | 86.53 | 23.18 | ^[26]^ |
| 2023 | 1.68 | 1.22 | 20.59 | 84.28 | 21.1 | ^[27]^ |
| 2023 | 1.68 | 1.24 | 21.16 | 82.5 | 21.63 | ^[28]^ |
| 2023 | 1.68 | 1.2 | 21.33 | 82.39 | 21.13 | ^[29]^ |
| 2023 | 1.68 | 1.18 | 20.43 | 83.33 | 20.09 | ^[30]^ |
| 2023 | 1.73 | 1.23 | 19.87 | 76.31 | 18.6 | ^[31]^ |
| 2023 | 1.73 | 1.21 | 20.33 | 83.6 | 20.6 | ^[32]^ |
| 2023 | 1.74 | 1.195 | 21.1 | 79 | 19.91 | ^[33]^ |
| 2023 | 1.74 | 1.27 | 20.23 | 78.07 | 20.12 | ^[34]^ |
| 2023 | 1.75 | 1.27 | 18.17 | 86.31 | 20 | ^[35]^ |
| 2023 | 1.75 | 1.29 | 18.3 | 85.2 | 20.1 | ^[36]^ |
| 2023 | 1.76 | 1.32 | 17.6 | 85.1 | 19.6 | ^[37]^ |
| 2023 | 1.77 | 1.33 | 17.75 | 82.7 | 19.53 | ^[38]^ |
| 2023 | 1.77 | 1.29 | 17.54 | 82.61 | 18.63 | ^[39]^ |
| 2023 | 1.77 | 1.32 | 18.22 | 83.08 | 19.85 | ^[40]^ |
| 2023 | 1.77 | 1.29 | 17.54 | 82.61 | 18.63 | ^[41]^ |
| 2023 | 1.78 | 1.36 | 17.65 | 83.11 | 19.83 | ^[42]^ |
| 2023 | 1.79 | 1.32 | 18.01 | 84.37 | 20.06 | ^[43]^ |
| 2023 | 1.8 | 1.34 | 18.2 | 83.9 | 20.3 | ^[44]^ |
| 2023 | 1.85 | 1.35 | 16.78 | 83.29 | 18.87 | ^[45]^ |
| 2022 | 1.63 | 1.18 | 22.56 | 81.76 | 21.76 | ^[46]^ |
|  | 1.68 | 1.21 | 20.98 | 79.45 | 20.11 |  |
| 2022 | 1.65 | 1.22 | 21.5 | 83.3 | 21.9 | ^[47]^ |
| 2022 | 1.65 | 1.2 | 22.15 | 83.81 | 22.3 | ^[48]^ |
| 2022 | 1.73 | 1.31 | 18.89 | 81.6 | 20.22 | ^[49]^ |
| 2022 | 1.74 | 1.25 | 20.03 | 81.22 | 20.37 | ^[50]^ |
| 2022 | 1.75 | 1.3 | 18.9 | 77.8 | 19.07 | ^[51]^ |
| 2022 | 1.79 | 1.33 | 17.3 | 83.9 | 19.3 | ^[52]^ |
| 2019 | 1.68 | 1.17 | 21.2 | 79.8 | 19.8 | ^[53]^ |
| **2024** | **1.80** | **1.285** | **18.36** | **85.39** | **20.14** | **This work** |

**References**

[1] C. Duan, H. Gao, K. Xiao, V. Yeddu, B. Wang, R. Lin, H. Sun, P. Wu, Y. Ahmed, A. D. Bui, X. Zheng, Y. Wang, J. Wen, Y. Wang, W. Ou, C. Liu, Y. Zhang, H. Nguyen, H. Luo, L. Li, Y. Liu, X. Luo, M. I. Saidaminov, H. Tan, *Nat. Energy* **2024**, DOI 10.1038/s41560-024-01672-x.

[2] R. Wang, J. Zhu, J. You, H. Huang, Y. Yang, R. Chen, J. Wang, Y. Xu, Z. Gao, J. Chen, B. Xu, B. Wang, C. Chen, D. Zhao, W.-H. Zhang, *Energy Environ. Sci.* **2024**, *17*, 2662.

[3] X. Huo, S. Mariotti, Y. Li, T. Guo, C. Ding, P. Ji, S. Yuan, T. Li, N. Meng, X. Liu, J. Zhang, I. N. Rabehi, Y. Zhang, S. Zhao, H. Wang, D. Song, L. K. Ono, Z. Xu, Y. Qi, *Energy Environ. Sci.* **2024**, *17*, 8658.

[4] T. Huang, F. Xu, J. Hu, J. Wu, S. Li, P. Chen, X. Jia, Q. Li, H. Yan, Y. Ji, D. Luo, D. Wang, J. Hu, H.-H. Chen, Z. Lu, H. Xu, L. Li, R. Sha, Q. Zhong, X. Bai, M. I. Dar, T. Song, Z. Li, X. Yang, L. Zhao, Z.-H. Lu, Q. Gong, R. Zhu, *Energy Environ. Sci.* **2024**, *17*, 5984.

[5] T. Nie, Z. Fang, T. Yang, K. Zhao, J. Ding, S. (Frank) Liu, *Angew. Chem. Int. Ed.* **2024**, *63*, e202400205.

[6] X. Ji, S. Zhang, F. Yu, H. Zhang, L. Zhan, Y. Hu, W.-H. Zhu, Y. Wu, *Sci. China Chem.* **2024**, *67*, 2102.

[7] Q. Ye, W. Hu, J. Zhu, Z. Cai, H. Zhang, T. Dong, B. Yu, F. Chen, X. Wei, B. Yao, W. Dou, Z. Fang, F. Ye, Z. Liu, T. Li, *Energy Environ. Sci.* **2024**, *17*, 5866.

[8] Q. Ma, Y. Wang, L. Liu, P. Yang, W. He, X. Zhang, J. Zheng, M. Ma, M. Wan, Y. Yang, C. Zhang, T. Mahmoudi, S. Wu, C. Liu, Y.-B. Hahn, Y. Mai, *Energy Environ. Sci.* **2024**, *17*, 1637.

[9] Y. Luo, J. Zhu, X. Yin, W. Jiao, Z. Gao, Y. Xu, C. Wang, Y. Wang, H. Lai, H. Huang, J. Luo, J. Wang, J. You, Z. Zhang, X. Hao, G. Zeng, S. Ren, Z. Li, F. Fu, M. Li, C. Xiao, C. Chen, D. Zhao, *Adv. Energy Mater.* **2024**, *14*, 2304429.

[10] A. Zhang, M. Li, C. Dong, W. Ye, X. Yang, A. Shaker, M. S. Salem, Z. Li, J. Yang, X. Li, L. Xu, H. Song, C. Chen, J. Tang, *Small* **2024**, *20*, 2401197.

[11] X. Jiang, Q. Zhou, Y. Lu, H. Liang, W. Li, Q. Wei, M. Pan, X. Wen, X. Wang, W. Zhou, D. Yu, H. Wang, N. Yin, H. Chen, H. Li, T. Pan, M. Ma, G. Liu, W. Zhou, Z. Su, Q. Chen, F. Fan, F. Zheng, X. Gao, Q. Ji, Z. Ning, *Natl. Sci. Rev.* **2024**, *11*, nwae055.

[12] Y. Guo, S. Guo, T. Wu, S. Zhan, C. Wei, X. Luo, J. Huang, J. Su, Y. Hua, B. Xu, *Chem. Eng. J.* **2024**, *497*, 154722.

[13] S. Wu, Y. Yan, J. Yin, K. Jiang, F. Li, Z. Zeng, S.-W. Tsang, A. K.-Y. Jen, *Nat. Energy* **2024**, *9*, 411.

[14] Z. Zhang, W. Chen, X. Jiang, J. Cao, H. Yang, H. Chen, F. Yang, Y. Shen, H. Yang, Q. Cheng, X. Chen, X. Tang, S. Kang, X. Ou, C. J. Brabec, Y. Li, Y. Li, *Nat. Energy* **2024**, *9*, 592.

[15] X. Guo, Z. Jia, S. Liu, R. Guo, F. Jiang, Y. Shi, Z. Dong, R. Luo, Y.-D. Wang, Z. Shi, J. Li, J. Chen, L. K. Lee, P. Müller-Buschbaum, D. S. Ginger, D. J. Paterson, Y. Hou, *Joule* **2024**, *8*, 2554.

[16] S. Li, Z. Zheng, J. Ju, S. Cheng, F. Chen, Z. Xue, L. Ma, Z. Wang, *Adv. Mater.* **2024**, *36*, 2307701.

[17] Y. An, N. Zhang, Z. Zeng, Y. Cai, W. Jiang, F. Qi, L. Ke, F. R. Lin, S.-W. Tsang, T. Shi, A. K.-Y. Jen, H.-L. Yip, *Adv. Mater.* **2024**, *36*, 2306568.

[18] A. Z. Afshord, B. E. Uzuner, W. Soltanpoor, S. H. Sedani, T. Aernouts, G. Gunbas, Y. Kuang, S. Yerci, *Adv. Funct. Mater.* **2023**, *33*, 2301695.

[19] P. Hang, C. Kan, B. Li, Y. Yao, Z. Hu, Y. Zhang, J. Xie, Y. Wang, D. Yang, X. Yu, *Adv. Funct. Mater.* **2023**, *33*, 2214381.

[20] Z. Li, X. Sun, X. Zheng, B. Li, D. Gao, S. Zhang, X. Wu, S. Li, J. Gong, J. M. Luther, Z. Li, Z. Zhu, *Science* **2023**, *382*, 284.

[21] G. Su, R. Yu, Y. Dong, Z. He, Y. Zhang, R. Wang, Q. Dang, S. Sha, Q. Lv, Z. Xu, Z. Liu, M. Li, Z. Tan, *Adv. Energy Mater.* **2024**, *14*, 2303344.

[22] J. Wen, Y. Zhao, P. Wu, Y. Liu, X. Zheng, R. Lin, S. Wan, K. Li, H. Luo, Y. Tian, L. Li, H. Tan, *Nat. Commun.* **2023**, *14*, 7118.

[23] Z. Song, J. Yang, X. Dong, R. Wang, Y. Dong, D. Liu, Y. Liu, *Nano Lett.* **2023**, *23*, 6705.

[24] H. Guan, S. Zhou, S. Fu, D. Pu, X. Chen, Y. Ge, S. Wang, C. Wang, H. Cui, J. Liang, X. Hu, W. Meng, G. Fang, W. Ke, *Adv. Mater.* **2024**, *36*, 2307987.

[25] L. Qiao, T. Ye, T. Wang, W. Kong, R. Sun, L. Zhang, P. Wang, Z. Ge, Y. Peng, X. Zhang, M. Xu, X. Yan, J. Yang, X. Zhang, F. Zeng, L. Han, X. Yang, *Adv. Energy Mater.* **2024**, *14*, 2302983.

[26] L. Liu, B. Farhadi, J. Li, S. Liu, L. Lu, H. Wang, M. Du, L. Yang, S. Bao, X. Jiang, X. Dong, Q. Miao, D. Li, K. Wang, S. F. Liu, *Angew. Chem. Int. Ed.* **2024**, *63*, e202317972.

[27] C. Li, Z. Zhang, H. Zhang, W. Yan, Y. Li, L. Liang, W. Yu, X. Yu, Y. Wang, Y. Yang, M. K. Nazeeruddin, P. Gao, *Angew. Chem. Int. Ed.* **2024**, *63*, e202315281.

[28] N. Yan, Y. Gao, J. Yang, Z. Fang, J. Feng, X. Wu, T. Chen, S. (Frank) Liu, *Angew. Chem. Int. Ed.* **2023**, *62*, e202216668.

[29] T. Nie, J. Yang, Z. Fang, Z. Xu, X. Ren, X. Guo, T. Chen, S. (Frank) Liu, *Chem. Eng. J.* **2023**, *468*, 143341.

[30] C. Su, R. Wang, J. Tao, J. Shen, D. Wang, L. Wang, G. Fu, S. Yang, M. Yuan, T. He, *J. Mater. Chem. A* **2023**, *11*, 6565.

[31] L. Yang, Y. Jin, Z. Fang, J. Zhang, Z. Nan, L. Zheng, H. Zhuang, Q. Zeng, K. Liu, B. Deng, H. Feng, Y. Luo, C. Tian, C. Cui, L. Xie, X. Xu, Z. Wei, *Nano Micro Lett.* **2023**, *15*, 111.

[32] T. Xu, W. Xiang, J. Yang, D. J. Kubicki, W. Tress, T. Chen, Z. Fang, Y. Liu, S. Liu, *Adv. Mater.* **2023**, *35*, 2303346.

[33] F. Bisconti, M. Leoncini, S. Gambino, N. Vanni, S. Carallo, F. Russo, V. Armenise, A. Listorti, S. Colella, S. Valastro, A. Alberti, G. Mannino, A. Rizzo, *ACS Nano* **2024**, *18*, 1573.

[34] Y. Yang, L. Liu, J. Li, S. Zhao, Z. Chang, L. Wang, D. Yu, K. Wang, S. (Frank) Liu, *Nano Energy* **2023**, *109*, 108288.

[35] J. Heo, S. W. Lee, J. Yong, H. Park, Y. K. Lee, J. Shin, D. R. Whang, D. W. Chang, H. J. Park, *Chem. Eng. J.* **2023**, *474*, 145632.

[36] J. Yong, Y. K. Lee, H. Park, S. Muthu, J. Shin, D. R. Whang, B.-G. Kim, D. W. Chang, H. J. Park, *Adv. Funct. Mater.* **2024**, *34*, 2312505.

[37] B. Niu, H. Liu, Y. Huang, E. Gu, M. Yan, Z. Shen, K. Yan, B. Yan, J. Yao, Y. Fang, H. Chen, C.-Z. Li, *Adv. Mater.* **2023**, *35*, 2212258.

[38] Z. Yi, W. Wang, R. He, J. Zhu, W. Jiao, Y. Luo, Y. Xu, Y. Wang, Z. Zeng, K. Wei, J. Zhang, S.-W. Tsang, C. Chen, W. Tang, D. Zhao, *Energy Environ. Sci.* **2024**, *17*, 202.

[39] W. Wang, X. Liu, J. Wang, C. Chen, J. Yu, D. Zhao, W. Tang, *Adv. Energy Mater.* **2023**, *13*, 2300694.

[40] H. Liu, J. Dong, P. Wang, B. Shi, Y. Zhao, X. Zhang, *Adv. Funct. Mater.* **2023**, *33*, 2303673.

[41] W. Wang, X. Liu, J. Wang, C. Chen, J. Yu, D. Zhao, W. Tang, *Adv. Energy Mater.* **2023**, *13*, 2300694.

[42] H. Cui, L. Huang, S. Zhou, C. Wang, X. Hu, H. Guan, S. Wang, W. Shao, D. Pu, K. Dong, J. Zhou, P. Jia, W. Wang, C. Tao, W. Ke, G. Fang, *Energy Environ. Sci.* **2023**, *16*, 5992.

[43] L. Qiao, T. Ye, P. Wang, T. Wang, L. Zhang, R. Sun, W. Kong, X. Yang, *Adv. Funct. Mater.* **2024**, *34*, 2308908.

[44] F. Yang, P. Tockhorn, A. Musiienko, F. Lang, D. Menzel, R. Macqueen, E. Köhnen, K. Xu, S. Mariotti, D. Mantione, L. Merten, A. Hinderhofer, B. Li, D. R. Wargulski, S. P. Harvey, J. Zhang, F. Scheler, S. Berwig, M. Roß, J. Thiesbrummel, A. Al-Ashouri, K. O. Brinkmann, T. Riedl, F. Schreiber, D. Abou-Ras, H. Snaith, D. Neher, L. Korte, M. Stolterfoht, S. Albrecht, *Adv. Mater.* **2024**, *36*, 2307743.

[45] X. Wang, D. Zhang, B. Liu, X. Wu, X. Jiang, S. Zhang, Y. Wang, D. Gao, L. Wang, H. Wang, Z. Huang, X. Xie, T. Chen, Z. Xiao, Q. He, S. Xiao, Z. Zhu, S. Yang, *Adv. Mater.* **2023**, *35*, 2305946.

[46] Y. Zheng, X. Wu, J. Liang, Z. Zhang, J. Jiang, J. Wang, Y. Huang, C. Tian, L. Wang, Z. Chen, C.-C. Chen, *Adv. Funct. Mater.* **2022**, *32*, 2200431.

[47] Z. Liu, C. Zhu, H. Luo, W. Kong, X. Luo, J. Wu, C. Ding, Y. Chen, Y. Wang, J. Wen, Y. Gao, H. Tan, *Adv. Energy Mater.* **2023**, *13*, 2203230.

[48] Z. Zhu, K. Mao, K. Zhang, W. Peng, J. Zhang, H. Meng, S. Cheng, T. Li, H. Lin, Q. Chen, X. Wu, J. Xu, *Joule* **2022**, *6*, 2849.

[49] Y. Zhao, C. Wang, T. Ma, L. Zhou, Z. Wu, H. Wang, C. Chen, Z. Yu, W. Sun, A. Wang, H. Huang, B. Zou, D. Zhao, X. Li, *Energy Environ. Sci.* **2023**, *16*, 2080.

[50] L. Liu, Y. Yang, M. Du, Y. Cao, X. Ren, L. Zhang, H. Wang, S. Zhao, K. Wang, S. (Frank) Liu, *Adv. Energy Mater.* **2023**, *13*, 2202802.

[51] Y. Yu, R. Liu, C. Liu, X.-L. Shi, H. Yu, Z.-G. Chen, *Adv. Energy Mater.* **2022**, *12*, 2201509.

[52] H. Chen, A. Maxwell, C. Li, S. Teale, B. Chen, T. Zhu, E. Ugur, G. Harrison, L. Grater, J. Wang, Z. Wang, L. Zeng, S. M. Park, L. Chen, P. Serles, R. A. Awni, B. Subedi, X. Zheng, C. Xiao, N. J. Podraza, T. Filleter, C. Liu, Y. Yang, J. M. Luther, S. De Wolf, M. G. Kanatzidis, Y. Yan, E. H. Sargent, *Nature* **2023**, *613*, 676.

[53] D. H. Kim, C. P. Muzzillo, J. Tong, A. F. Palmstrom, B. W. Larson, C. Choi, S. P. Harvey, S. Glynn, J. B. Whitaker, F. Zhang, Z. Li, H. Lu, M. F. A. M. van Hest, J. J. Berry, L. M. Mansfield, Y. Huang, Y. Yan, K. Zhu, *Joule* **2019**, *3*, 1734.
